# DockAnalyse: an application for the analysis of protein-protein interactions

**DOI:** 10.1186/1472-6807-10-37

**Published:** 2010-10-22

**Authors:** Isaac Amela, Pedro Delicado, Antonio Gómez, Sílvia Bonàs, Enrique Querol, Juan Cedano

**Affiliations:** 1 Institut de Biotecnologia i de Biomedicina Parc de Recerca UAB and Departament de Bioquímica i Biologia Molecular, Universitat Autònoma de Barcelona. 08193-Bellaterra, Barcelona, Spain; 2 Departament d'Estadística i Investigació Operativa, Universitat Politècnica de Catalunya. 08034-Barcelona, Spain

## Abstract

**Background:**

Is it possible to identify what the best solution of a docking program is? The usual answer to this question is the highest score solution, but interactions between proteins are dynamic processes, and many times the interaction regions are wide enough to permit protein-protein interactions with different orientations and/or interaction energies. In some cases, as in a multimeric protein complex, several interaction regions are possible among the monomers. These dynamic processes involve interactions with surface displacements between the proteins to finally achieve the functional configuration of the protein complex. Consequently, there is not a static and single solution for the interaction between proteins, but there are several important configurations that also have to be analyzed.

**Results:**

To extract those representative solutions from the docking output datafile, we have developed an unsupervised and automatic clustering application, named *DockAnalyse*. This application is based on the already existing DBscan clustering method, which searches for continuities among the clusters generated by the docking output data representation. The DBscan clustering method is very robust and, moreover, solves some of the inconsistency problems of the classical clustering methods like, for example, the treatment of outliers and the dependence of the previously defined number of clusters.

**Conclusions:**

*DockAnalyse *makes the interpretation of the docking solutions through graphical and visual representations easier by guiding the user to find the representative solutions. We have applied our new approach to analyze several protein interactions and model the dynamic protein interaction behavior of a protein complex. *DockAnalyse *might also be used to describe interaction regions between proteins and, therefore, guide future flexible dockings. The application (implemented in the R package) is accessible.

## Background

Protein-protein interaction (PPI) is the key process by which most of the proteins fulfill their function, and interactomics represents one of the current frontiers of biosciences [1,2]. It is well known that many proteins are single parts, called monomers, of a complex quaternary structure, a multimer. In any case, monomers alone do not have a specific function which is only achieved when the distinct parts interact together to accomplish a certain function [3,4]. PPIs can help us to predict protein function and, therefore, many protein function predictors have been developed using PPI databases [5-11]. Due to PPIs, it is expected that in the near future the number of protein complexes will surpass the number of proteins in some organisms. A lot of PPIs involve surface displacements among the members of the protein complex to achieve the required biological function.

Nuclear Magnetic Resonance (NMR) and X-Ray Crystallography (XRC) are the two main technologies applied for structure elucidation, but these hi-tech methods are frequently constrained by the methodological requirements when dealing with protein complexes. It is assumed that these experimental limitations have reduced the amount of large protein complexes solved and, therefore, protein complexes have become less represented in the structural databases such as the Protein Data Bank (PDB; http://www.rcsb.org/pdb/home/home.do; [12]). Therefore, when trying to analyze the dynamics of the interaction process among the proteins of a protein complex, a NMR spectroscopic technique may not be feasible, and the data obtained of a XRC experiment may not be useful to represent the dynamic behavior. Consequently, despite the use of these two experimental technologies for protein structure determination being widely distributed, other complementary strategies may be useful to accurately model the dynamics of the interaction among the proteins of a protein complex. In this context, some theoretical methods to study protein complexes at a structural level, such as docking, are now emerging. Protein-protein docking (PPD) is a computational method to predict the best way by which two proteins could interact [13,14]. In rigid body PPD approaches, conformational changes during the complex formation are not permitted, in order to save computation time. This technique may be appropriate when non-substantial conformational changes are expected to take place in the interacting proteins.

Usually, it is considered that the best solution given by a docking program is the one with the best interaction energy, but quite a lot of the real interactions tend to involve large surface displacements with non-optimal interaction energies to finally form the protein complex. These displacements occur along the protein surface, generating multiple low-energy interaction complexes. In these cases, these low-energy interaction regions might not be, in reality, less important from a functional point of view, and the interaction region has to be wide enough to allow PPIs coming from different orientations like, for instance, proteins that require movements among them when they act as a protein complex. Owing to all of these facts, interaction among proteins seems to be a dynamic mechanism where there is not only one single solution with the best interaction energy, like most of the current PPD programs consider, but rather there are several solutions with more or less interaction energy, and not necessarily does the native form have the best theoretical solution [15].

Our approach attempts to deal with these particularities by considering the global contribution of the PPD calculated solutions and selecting those representative solutions (docking solutions that are centers of clusters and have high interaction energy) that describe a general behavior of a subset of solutions without improving an unrealistic one. To extract those solutions that best describe the real dynamic mechanism of interaction from the output datafile of the current PPD programs, we have developed an application, called *DockAnalyse*, which is based on the already-existing DBscan clustering method [16] and, moreover, is unsupervised and automatic. The aim of this new method is to choose the appropriate solutions, not only by taking into account the interaction energy, but also the dependence among the clusters generated by the docking outputdata representation. The way of choosing the representative solutions is made by searching for continuities among these clusters. The real challenge of the newly developed application is the ability to identify significant structures from the huge amount of previously calculated docking solutions without requiring too many tuning parameters from the user in order to run the program. Normally, the decision about which of the docked structures is the most important is very difficult, but *DockAnalyse *guides the search for good docking candidates by reducing the huge amount of putative docking solutions to check. Furthermore, the use of *DockAnalyse *allows a global vision of many characteristics of the PPI process through different data, graphical representations and possible personalized searches which also guides the search for signifiant solutions.

The exhaustive analysis of all of the PPI structures obtained with *DockAnalyse *may help us to theoretically postulate the structure of the studied protein complex or to propose the way by which certain proteins interact together in a mobile fashion to execute a biological function. Moreover, the analysis made by *DockAnalyse *might guide future flexible PPD approaches, because of the important PPD information obtained from the use of this new application.

## Results and Discussion

### Characteristics of DockAnalyse

With the aim of elucidating which of the docked structures between two studied protein structures are the most important from a functional point of view, an unsupervised procedure, based on the already existing DBscan clustering method [16], was designed and implemented with the R package. The movement, expressed in rotations and translations described by the proteins, and the interaction energy were considered in the algorithm to finally obtain the cluster distribution with the best internal coherence among the clusters generated by the docking output datafile representation. An initial transformation of the angles is required in order to make them comparable to location information.

First, distances among angles of the different docking solutions are computed. For instance, assume that *A *= (*a*_1_, *a*_2_, *a*_3_) and *B *= (*b*_1_, *b*_2_, *b*_3_) are the angles corresponding to two docking solutions, then the distance between A and B is defined as:

$$d\left(A,\;B\right)=\sqrt{d_{1}^{2}+d_{2}^{2}+d_{3}^{2}}$$

where *d_i _*= min{|*b_i _*- *a_i_*|, |*b_i _*- *a_i _*+ 2π|, |*b_i _*- *a_i _*= 2π|} for *i *= 1, 2, 3.

That is, *d_i _*is the angular distance between angles *a_i _*and *b_i_*. Once the distance matrix between angles of docking solutions has been computed, Multidimensional Scaling (MDS) [17] is performed on this distance matrix. The resulting principal coordinates are then concatenated to position variables in the docking output datafile in order to have a new data matrix where the Euclidean distances between rows represent the joint distance between angles and location of docking solutions. Moreover, the weight (or variability) and the information of each angle/position are forced to be the same in the new data matrix. Then, an automatic pre-processing step finds the radius necessary to run the DBscan clustering method, and the best ε (density reachability distance) parameter possible, also necessary for a proper DBscan analysis, is chosen according to a battery of cluster quality measures. To be specific, high values for high quality clustering of the following indexes have been considered (See Walesiak and Dudek (2007) [18] for more details): Davies-Bouldin (multiplied by -1), Calinski-Harabasz, Hubert-Levine (multiplied by -1) and Silhouette. DBscan is applied to several ε-candidate values and the resulting clusters are evaluated by these criteria. The ε-candidate values are ranked according to every index, and the score of an ε-candidate value is then established as the mean of its ranks. Finally, the value with the highest score is taken as the final ε and the corresponding cluster is considered to be the right one.

**Figure 1:**
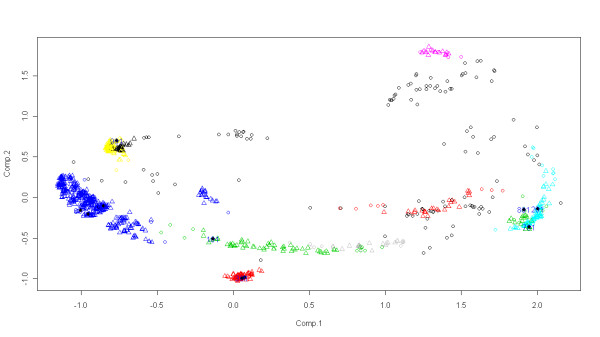
**Example of one of the *DockAnalyse *graphical ouput windows of a certain docking assay**. This is one of the most important output windows of *DockAnalyse*, which shows the clustering graph of all of the docking solutions tested. The axes are the two extracted components of the computed Principal Components Analysis (PCA). The clusters found by the program are depicted in different colors, and the representative points of each cluster are highlighted. This, and all of the other *DockAnalyse *output representations, allow for an easy and visual interpretation of the docking procedure.

*DockAnalyse*, was applied to interpret the results obtained from different PPD assays because it gives a lot of information that is produced by the PPD output datafile analysis. As well as this information, the shape, size and distribution of the clusters obtained along with the position of the outliers are shown in *DockAnalyse *result graphical representations (See Figure 1 for an example). These graphs give a global vision of the PPI process, enabling a curated study of the most interesting PPD solutions. Figure 1 represents a bidimensional graph that depicts the representation in a plane of a cloud of points in a multidimensional space (here 8 dimensions) after the application of a method to reduce dimensions (in this case Principal Component Analysis [19]). As is well-known, the two first principal components explain the main part of the variability contained in the data, but usually it is impossible to reflect all of the distances among the points in a multidimensional space in only two dimensions, which is why outlier points might appear very close to other cluster centers. To check that point, the alternative representation in the script where all combinations of axes are depicted can be activated (See the "readme" file of the application). The representative solutions of each of the calculated clusters can be highlighted and they refer to the significant points among all of the docked structures tested (See, again, Figure 1). These points represent the most relevant solutions obtained from the PPD calculation and they allow us to identify which solutions among all might be more directly involved in the PPI process. These representatives (or representative solutions) are central members of the clusters and they also have high interaction energy. What represents a real challenge, which is facilitated by *DockAnalyse*, is the reduction of the number of solutions to analyze after the PPD experiment. With our program, the docking outputdata analysis is facilitated because the number of solutions is reduced from a huge number (e.g., 1000) to approximately less than 10 in most of the cases. Some PPD programs do not incorporate a clustering process and the use of DockAnalyse in these cases is even more justified. Evidently, *DockAnalyse *gives researchers the possibility to use it with a greater or lesser number of docking solutions although this characteristic has been proposed to guarantee the exhaustive exploration of the whole space of docking solutions.

**Figure 2:**
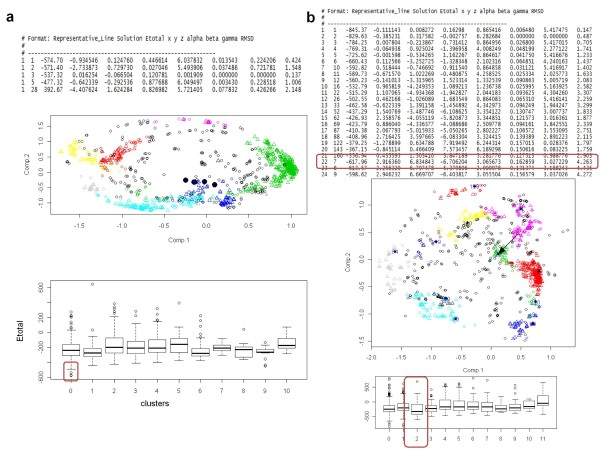
**Initially low-scored docking solutions might be important and considered with the use of *DockAnalyse***. (a) Firstly, the best solutions of the original results of the PPD program with each RMS deviation values calculated are shown. Below, these optimal solutions are highlighted in the graphical representations provided by *DockAnalyse*. These configurations can be interpreted as a binding site with a high level of constraints that seem to describe an interaction pocket. Despìte not belonging to any representative cluster, their high interaction energies reveal this type of static contact. (b) A section of the raw output datafile of the PPD program as well as two of the graphical representation outputs obtained with *DockAnalyse *for these same data are depicted. The most representative *DockAnalyse *solution is highlighted in the three sections showing the ability of this new application to consider important alternative solutions.

The main advantage of *DockAnalyse*, when trying to interpret the results from a docking procedure, is shown in Figure 1 and in Figure 2, where it can be seen that obtaining conclusions from the graphical representations given by *DockAnalyse *is much more intuitive than from the raw numerical data given by most of the PPD programs.

### Testing DockAnalyse

Through a set of 35 Enzyme/Inhibitor or Enzyme/Substrate protein complexes of the current Protein-Protein Docking Benchmark 3.0 [20] (which are labeled with an "E" in the benchmark table), we have shown the way by which *DockAnalyse *can be applied in a systematic way to monitor the quality and type of docking predictions. This group of known protein complexes included homodimers and heterodimers, protein inhibitors and other enzyme complexes. The unbound structures of the interacting proteins were used when available; otherwise, the bound structures were extracted from the complex.

The percentage of satisfactory dockings detected was 51.43%, where a satisfactory docking is the one on which one or more *DockAnalyse *clusters are significant in terms of a high number of members and high average interaction energy (These can be easily seen through *DockAnalyse *graphical outputs). In comparison to the crystallographic protein complex structure, which was obtained from the benchmark set, all of these satisfactory solutions showed a very low RMS (Root Mean Square) deviation. This means that only through *DockAnalyse *outputs could it be seen in these cases that the dockings were credible before realizing that the RMS deviation was so low. On the contrary, the percentage of unsatisfactory dockings detected was 28.57%, where an unsatisfactory docking means that all of the clusters given by *DockAnalyse *are composed of few members and, moreover, have very low interaction energies. The RMS deviation values calculated here were all very high. Again, with *DockAnalyse *these unsatisfactory dockings could be detected before knowing their high RMS deviation values. RMS deviation values could be calculated because we knew the crystallographic structure of the protein complex from the benchmark, but in real research it will almost never be known, so *DockAnalyse *might be used at this point to guide the researcher concerning the quality and credibility of the docking. In addition, 17.14% of the dockings were considered to be static interactions with a RMS deviation again very low in the considered solutions. The way by which *DockAnalyse *can detect this type of static interactions is explained below.

These interactions could be detected with our program due to the possibility to perform personalized searches, introducing specific PPD solution values in the "marker" variable of the program source code (See the "readme" file of the program). In Figure 2a, some of the best solutions of the docking program (with optimal RMS deviation values and good interaction energies) are interpreted by *DockAnalyse *as a clear trajectory. These values do not belong to any cluster so, consequently, they are included initially in Cluster 0, but the graphical representations provided by this tool and the different information given could help the user to realize that he is being faced with two proteins with a small binding site and without any permitted flexibility.

As has been reported thoroughly in previous sections, the main potential of this new method is the capability to explore the interaction space, making clusters that correspond to extensive contact regions. These graphical representations reproduce the movements that occur between the constituents of a protein complex. These preassumed non-optimal solutions described before have lower scores in the PPD program outputfile, therefore, they are discarded by the PPD initial filter. As shown below in the example, these solutions could be rescued and their score improved with *DockAnalyse *application. In Figure 2b, Solution 7 has been included in Cluster 2 in *DockAnalyse *results, but in the PPD program it is ranked as Solution 22, far from the optimal solution although both RMS deviation and energy values are significant. It has to be considered that *DockAnalyse *highlights this solution as one of the most representative because it is at the center of the cluster with the highest interaction energy (Cluster 2). Moreover, as can also be seen in Figure 2b, this cluster is in a highly connected interaction zone, demonstrating displacements among the two docked proteins. These are the types of results that could be obtained using *DockAnalyse*.

**Figure 3:**
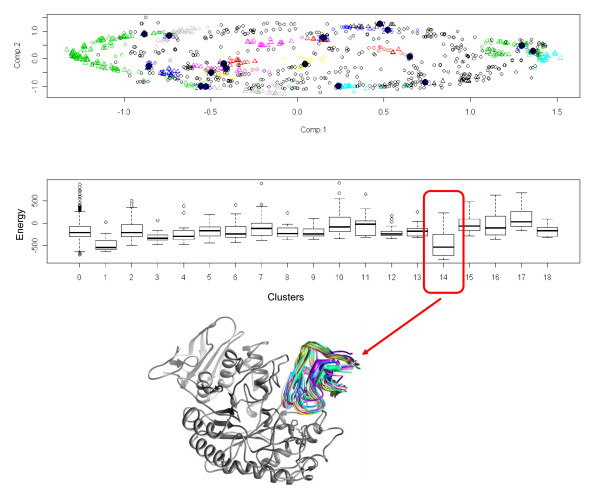
**Tridimensional visualization of the selected cluster**. One of the graphical results obtained with *DockAnalyse *for the protein complex of PDB: 1BVN of the Protein-Protein Docking Benchmark. Cluster 14 is significant in terms of cluster members and average interaction energy. All of the ligand structures of the previously selected cluster (depicted in different colors and displayed in the "ribbons" format) are viewed in 3 D on the receptor (depicted in gray and displayed in the "ribbons" format).

For Protein Complex 3 (PDB: 1BVN) of the benchmark, *DockAnalyse *outputs showed a satisfactory docking in which Cluster 14 was significant. Using the supplementary scripts that come with *DockAnalyse*, all of the ligand positions of the solutions of Cluster 14 were extracted as PDB files and then loaded in a protein modeling and visualization tool with the structure of the receptor. As can be seen in Figure 3, all of the ligand positions contained in this cluster were very similar and, therefore, corroborated the robustness of *DockAnalyse*. Furthermore, that is another useful way to apply our program.

### Modeling a Protein Complex

**Figure 4:**
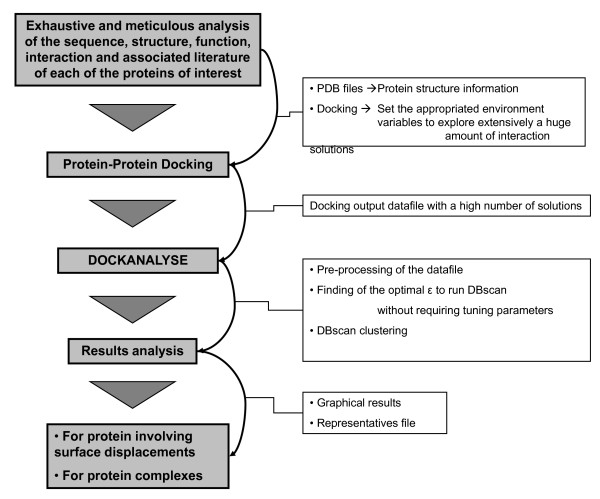
**Example of a protein complex modeling**. The images (a) -> (b) -> (c) -> (d) represent the modeled structures of the solutions given by *DockAnalyse *for the docking between proteins Isu1 and Isu2. The structures are displayed in the "surface" and "ribbon" formats and colored in green for protein Isu1 and yellow in the case of Isu2. The iron binding pocket of each of the proteins, which is composed of 3 cysteine residues, is displayed in a "ball and stick" format and colored in magenta. Isu1 and Isu2 iron binding pockets and interaction tails are labeled. The edges attempt to show the trajectory that may occur when these proteins interact to finally acquire the desired configuration required for ISC biogenesis.

An example of a procedure where *DockAnalyse *can be applied to model the movements between the members of a protein complex is as follows (This might be similar, in general.):

Isu1 and Isu2 are two yeast mitochondrial proteins which perform a scaffolding function during the assembly/maturation of Iron-Sulfur Cluster (ISC) prosthetic groups [20,21]. These proteins physically and functionally interact, leading to the formation of a stable protein complex [22]. To achieve the appropriate orientation between these two proteins, we have seen that Isu2 comes into contact with Isu1 and slips on it with the aim of reaching a certain orientation. In this appropriate position, the two proteins are situated one in front of the other and their tails might allow for the required stable interaction. Moreover, in this final conformation, 3 cysteine residues per protein (which typically conform an iron binding pocket) remain close enough to each other to be crucial for anchoring the ISC that is being generated while Isu1 and Isu2 tails facilitate their interaction [23] (Figure 4). Most of the studies prompt the suggestion that the iron and sulfur atoms required for the ISC biogenesis on Isu1/Isu2 are donated by other proteins, named Frataxin and Nfs1 [20]. This ISC biogenesis machinery is not yet well understood and problems in it cause several human diseases linked to protein/enzyme deficits. That is why the study of this prosthetic group generation represents an important challenge from any point of view. The sequence, structure, function and current literature of these proteins were analyzed in-depth. After that, PPD experiments were performed between the structures of the two proteins, setting up a small rotation step to exhaustively explore a great number of solutions in a reasonable computing time. Finally, *DockAnalyse *was applied with the aim of reducing the huge amount of docking solutions obtained to several representative ones. These solutions were the 4^th^, 78^th^, 28^th^, and 1^st ^initially ranked solutions of the Escher NG docking output datafile. Here, the main utility of *DockAnalyse *in reducing the number of solutions to analyze after a PPD calculation is shown. For these four representative docking solutions, the protein structure (PDB) files were obtained, merged into a trajectory file and then subsequently loaded into a protein modeling and visualization tool with which we could analyze them. This procedure allowed us to build a point-to-point pseudo-trajectory with which we could postulate a model to explain the surface displacements between the given proteins (Figure 4). This pseudo-trajectory could be reconstructed by means of the selection of other solutions along *DockAnalyse *clusters or by joining the different *DockAnalyse *cluster representatives. For this reason, the representative solutions could be considered to be static frames that describe the motion between the interacting proteins, and we could model/study the surface displacements of one protein on the other.

## Conclusions

A comprehensive tool for the analysis of PPIs has been designed. This new application permits a better interpretation of the obtained PPD solutions as well as the surface displacements that may occur during the interaction between the proteins of a protein complex. Therefore, this tool guides the modeling of a protein complex and can be applied in a systematic way to monitor the quality and type of docking predictions through global or local visions of the docking results that facilitate the desicion making process regarding the docking characteristics. The simplicity in applying this tool and the ease in interpreting the PPD solutions makes it ideally suited to analyze the data obtained in a PPD experiment. Considering all of the facts stated above, to go further and propose new functional interpretations for the proteins of interest might be much easier. In terms of these new hypotheses, when the initially docked proteins are monomers, a proposal on the putative structure of a multimeric protein complex might be postulated [3,4]. Another procedure to visualize the expected surface displacements between two interacting proteins may be suggested. This last approach could be applied to pairs of proteins that require displacements between them to fulfill a specific function [20].

**Figure 5:**
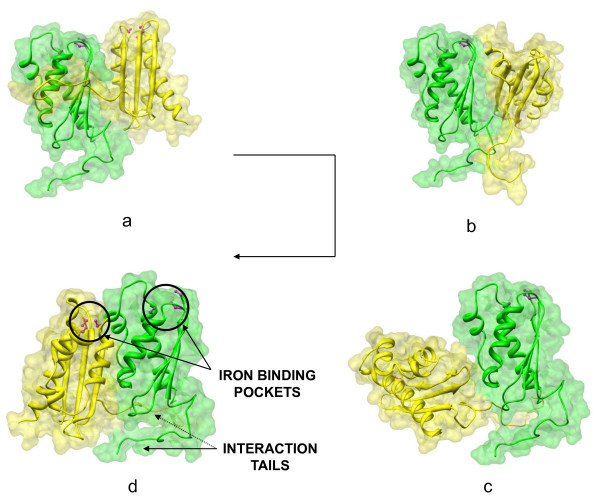
**DockAnalyse: how and when**. Schematic flowchart where the sequential steps of the bioinformatics study in which *DockAnalyse *might be used are described.

As a whole, *DockAnalyse *could be used after a docking assay in the context of a more complex procedure where a model of the behavior between the proteins that take part in a biologically functional protein complex would be performed. A schematic description of how to use *DockAnalyse *in this whole bioinformatics procedure is shown in Figure 5. First of all, an extensive literature mining analysis coupled with a profound study of the sequence, structure, function and interactome of the proteins of interest is required. Secondly, the required PPD experiments have to be executed, taking into account that the more solutions tested during the docking assays, the more robust the results from *DockAnalyse *would be. After that, the newly developed algorithm has to be applied to each of the docking output datafiles to obtain the representative docking solutions among those thousands calculated. Lastly, manual curation of the obtained docking representatives might be necessary to fit the solutions given with the appropriate biological function and to eliminate the putative aberrant results. The combination of theoretical docking procedures with the available experimental information is shown to greatly improve the modeling. *DockAnalyse *is accessible at: http://bioinf.uab.es/rker/DockAnalyse/DockAnalyse.zip (or see additional file 1: DockAnalyse).

## Methods

### Bases of DockAnalyse

The clustering algorithm used in the design of *DockAnalyse *was DBscan [16]. It has not been previously used for this purpose, it relies on a density based notion of clusters and it is designed to discover the clusters of arbitrary shape as well as to distinguish noise. The algorithm is based on the definition of density connection, where two points in a dataset are density connected if a chain of points in the dataset that allows for the movement from one to the other exists. The connecting chain must verify two conditions: firstly, each point of the chain (except, probably, the first and the last one) has at least k observed data at a distance less than a determined radius (ε), and, secondly, the distance between two consecutive points in the chain is less than ε. This definition induces a partition in the set of observed points and, therefore, each group is defined as a subset of points which are density connected among each other. The definitive clusters provided by DBscan are those components in the partition with, at least, two or more elements. DBscan considers those non-density connected points as isolated ones (groups with only one member or outliers).

DBscan has been designed to discover clusters of arbitrary shape due to the fact that it is a density based algorithm. It identifies dense regions (clusters) which are separated by regions of low density (considered as outliers). The lack of an appropriate outlier is a well-known weakness of one of the classical clustering methods like k-means, where even very far points from the closest centroid are included in the same cluster without any additional criteria. This clustering method was chosen because it is extremely robust and it also solves some inconsistency problems that usually appear when applying other clustering methods. In general, the classical clustering methods do not manage the outliers well, while DBscan tends to treat these isolated points much better and it allows for the finding of all cluster members independently of the cluster shape, discarding the outliers. One of the main problems of clustering is that the classical methods are dependent on the previously defined number of clusters, while DBscan is not.

The DBscan algorithm depends on two tuning parameters that define the density connection (k and εgreek small letter epsilon). Ester et al. (1996) [16] indicate that the choice of a good εgreek small letter epsilon is much more important than the choice of k. The results in their databases are quite similar for any k ≥ 4 and, therefore, they propose to fix k = 4. In our experiments, better results for k values greater than 4 were verified and, consequently, k = 15 was used in all computations. The classical purpose of executing the DBScan algorithm is usually to clusterize a cloud of points in order to group all of the points together according to their similarities, but in *DockAnalyse *we also used it to sort these clusters taking into account the number of members of each cluster. The more members a cluster has the better the solution is. This property allows for the sorting of the solution according to the number of members/solutions per cluster, and at the same time it allows for the removing of non-relevant docking solutions, which is the objective of fixing our k = 15 (minimum number of points in a cluster). The best solutions, which are the only ones that will be checked by the user of *DockAnalyse*, are integrated by hundreds of points. Those clusters with less than 15 members are discarded, therefore, they have no effect on the final result. This is done because nobody is going to check a docking representative with such a limited number of docking solutions supporting the goodness of this region to be a binding surface. Besides, the user can always access the script source code to modify the k parameter (minimum number of points in a cluster), but this is only an optional possibility because, as previously commented, this value has no relevant effect on the final results.

### Protein-Protein Dockings

*DockAnalyse *is currently designed to be used with whatever version of Escher NG or Hex [24-26] PPD programs, but by modifying only a few parameters of the script source code most of the PPD programs could also be employed. The only premise is that the PPD program must generate an output datafile composed of a matrix with information of rotations and translations for each solution. Despite the existence of the possibility of reducing the number of given solutions in the PPD experiment for most PPD programs, it has to be considered that the more solutions obtained in the docking assay, the more robust the *DockAnalyse *results would be.

## Authors' contributions

IA participated in the design of *DockAnalyse *and its use, performed the analysis, and wrote the manuscript. JC designed the use of the new tool, participated in the analysis and drafted the manuscript. PD and JC developed the new application in R. EQ and JC conceived of the study, participated in its coordination and helped to draft the manuscript. AG helped to analyze some data and drafted the manuscript. SB tested the new application. All authors read and approved the final manuscript.

## Acknowledgements and Funding

This research was supported by Grants (BIO2007-67904-C02-01, MTM2009-13985-C02-01, MTM2010-14887) from the MCYT (Ministerio de Ciencia y Tecnología, Spain), from the Centre de Referència de R+D de Biotecnologia de la Generalitat de Catalunya and from Fundació La Marató de TV3. EQ is Chair-Holder of a “Chair of Knowledge and Technology Transfer Parc de Recerca UAB- Santander”. AG acknowledges a postdoctoral fellowship from “Parc de Recerca UAB- Santander”. IA is a predoctoral fellowship recipient from the UAB (Universitat Autònoma de Barcelona). The English of this manuscript has been corrected by Mr. Chuck Simmons, a native English-speaking Instructor of English of this University.

## Supplementary Material

Additional file 1 **DockAnalyse**. 4 R program files, 4 Perl scripts, 2 text files and 3 zippped libraries. Click here for file
