# Supplementary material for: DockAnalyse: an application for the analysis of protein-protein interactions
Source: BMC Struct Biol. 2010 Oct 22;10:37. doi: 10.1186/1472-6807-10-37 (PMC2987812; doi:10.1186/1472-6807-10-37)
Supplement: Additional file 1 — DockAnalyse. 4 R program files, 4 Perl scripts, 2 text files and 3 zippped libraries. [file 1472-6807-10-37-S1.ZIP › ade4_1.4-14.zip!/ade4/html/00Index.html]

R: Analysis of Ecological Data : Exploratory and Euclidean methods
in Environmental sciences

# Analysis of Ecological Data : Exploratory and Euclidean methods in Environmental sciences


---

## Documentation for package ‘ade4’ version 1.4-14

- DESCRIPTION file.

## Help Pages

A
B
C
D
E
F
G
H
I
J
K
L
M
N
O
P
Q
R
S
T
U
V
W
Y
Z
misc

## -- --

|  |  |
| --- | --- |
| ade4-package | The ade4 package |

## -- A --

|  |  |
| --- | --- |
| abouheif.eg | Phylogenies and quantitative traits from Abouheif |
| acacia | Spatial pattern analysis in plant communities |
| acm.burt | Multiple Correspondence Analysis |
| acm.disjonctif | Multiple Correspondence Analysis |
| add.position.triangle | Triangular Plotting |
| add.scatter | Add graphics to an existing plot |
| add.scatter.eig | Add graphics to an existing plot |
| ade4 | The ade4 package |
| ade4toR | Format Change Utility |
| aminoacyl | Codon usage |
| amova | Analysis of molecular variance |
| apis108 | Allelic frequencies in ten honeybees populations at eight microsatellites loci |
| apqe | Apportionment of Quadratic Entropy |
| ardeche | Fauna Table with double (row and column) partitioning |
| area.plot | Graphical Display of Areas |
| area.util.class | Graphical Display of Areas |
| area.util.contour | Graphical Display of Areas |
| area.util.xy | Graphical Display of Areas |
| area2link | Graphical Display of Areas |
| area2poly | Graphical Display of Areas |
| arrival | Arrivals at an intensive care unit |
| as.data.frame.kdist | the class of objects 'kdist' (K distance matrices) |
| as.dudi | Duality Diagram |
| as.krandtest | Class of the Permutation Tests (in C). |
| as.randtest | Class of the Permutation Tests (in C). |
| as.rtest | Class of the Permutation Tests (in R). |
| as.taxo | Taxonomy |
| atlas | Small Ecological Dataset |
| atya | Genetic variability of Cacadors |
| avijons | Bird species distribution |
| avimedi | Fauna Table for Constrained Ordinations |
| aviurba | Ecological Tables Triplet |

## -- B --

|  |  |
| --- | --- |
| bacteria | Genomes of 43 Bacteria |
| banque | Table of Factors |
| baran95 | African Estuary Fishes |
| between | Between-Class Analysis |
| betweencoinertia | Between-class coinertia analysis |
| bf88 | Cubic Ecological Data |
| bicenter.wt | Double Weighted Centring |
| bordeaux | Wine Tasting |
| boxplot.acm | Multiple Correspondence Analysis |
| bsetal97 | Ecological and Biological Traits |
| buech | Buech basin |
| butterfly | Genetics-Ecology-Environment Triple |

## -- C --

|  |  |
| --- | --- |
| c.kdist | the class of objects 'kdist' (K distance matrices) |
| c.ktab | the class of objects 'ktab' (K-tables) |
| cailliez | Transformation to make Euclidean a distance matrix |
| capitales | Road Distances |
| carni19 | Phylogeny and quantative trait of carnivora |
| carni70 | Phylogeny and quantitative traits of carnivora |
| carniherbi49 | Taxonomy, phylogenies and quantitative traits of carnivora and herbivora |
| casitas | Enzymatic polymorphism in Mus musculus |
| cca | Canonical Correspondence Analysis |
| char2genet | A class of data: tables of populations and alleles |
| chatcat | Qualitative Weighted Variables |
| chats | Pair of Variables |
| chazeb | Charolais-Zebus |
| chevaine | Enzymatic polymorphism in Leuciscus cephalus |
| clementines | Fruit Production |
| cnc2003 | Frequenting movie theaters in France in 2003 |
| coinertia | Coinertia Analysis |
| col.names | the class of objects 'ktab' (K-tables) |
| col.names.ktab | the class of objects 'ktab' (K-tables) |
| col.names<- | the class of objects 'ktab' (K-tables) |
| col.names<-.ktab | the class of objects 'ktab' (K-tables) |
| coleo | Table of Fuzzy Biological Traits |
| combine.4thcorner | Functions to compute the fourth-corner statistic |
| corkdist | Tests of randomization between distances applied to 'kdist' objetcs |
| corvus | Corvus morphology |
| costatis | STATIS and Co-Inertia : Analysis of a series of paired ecological tables |
| count2genet | A class of data: tables of populations and alleles |

## -- D --

|  |  |
| --- | --- |
| deug | Exam marks for some students |
| disc | Rao's dissimilarity coefficient |
| discrimin | Linear Discriminant Analysis (descriptive statistic) |
| discrimin.coa | Discriminant Correspondence Analysis |
| dist.binary | Computation of Distance Matrices for Binary Data |
| dist.dudi | Computation of the Distance Matrix from a Statistical Triplet |
| dist.genet | Genetic distances from gene frequencies |
| dist.ktab | Mixed-variables coefficient of distance |
| dist.neig | Computation of the Distance Matrix associated to a Neighbouring Graph |
| dist.prop | Computation of Distance Matrices of Percentage Data |
| dist.quant | Computation of Distance Matrices on Quantitative Variables |
| dist.taxo | Taxonomy |
| divc | Rao's diversity coefficient also called quadratic entropy |
| divcmax | Maximal value of Rao's diversity coefficient also called quadratic entropy |
| dotchart.phylog | Representation of many quantitative variables in front of a phylogenetic tree |
| dotcircle | Representation of n values on a circle |
| doubs | Pair of Ecological Tables |
| dpcoa | Double principal coordinate analysis |
| dudi | Duality Diagram |
| dudi.acm | Multiple Correspondence Analysis |
| dudi.coa | Correspondence Analysis |
| dudi.dec | Decentred Correspondence Analysis |
| dudi.fca | Fuzzy Correspondence Analysis and Fuzzy Principal Components Analysis |
| dudi.fpca | Fuzzy Correspondence Analysis and Fuzzy Principal Components Analysis |
| dudi.hillsmith | Ordination of Tables mixing quantitative variables and factors |
| dudi.mix | Ordination of Tables mixing quantitative variables and factors |
| dudi.nsc | Non symmetric correspondence analysis |
| dudi.pca | Principal Component Analysis |
| dudi.pco | Principal Coordinates Analysis |
| dunedata | Dune Meadow Data |

## -- E --

|  |  |
| --- | --- |
| ecg | Electrocardiogram data |
| ecomor | Ecomorphological Convergence |
| EH | Amount of Evolutionary History |
| elec88 | Electoral Data |
| enum.phylog | Plot phylogenies |
| escopage | K-tables of wine-tasting |
| euro123 | Triangular Data |

## -- F --

|  |  |
| --- | --- |
| fission | Fission pattern and heritable morphological traits |
| foucart | K-tables Correspondence Analysis with the same rows and the same columns |
| fourthcorner | Functions to compute the fourth-corner statistic |
| fourthcorner2 | Functions to compute the fourth-corner statistic |
| freq2genet | A class of data: tables of populations and alleles |
| friday87 | Faunistic K-tables |
| fruits | Pair of Tables |
| fuzzygenet | Reading a table of genetic data (diploid individuals) |

## -- G --

|  |  |
| --- | --- |
| gearymoran | Moran's I and Geary'c randomization tests for spatial and phylogenetic autocorrelation |
| genet | A class of data: tables of populations and alleles |
| ggtortoises | Microsatellites of Galapagos tortoises populations |
| granulo | Granulometric Curves |
| gridrowcol | Complete regular grid analysis |

## -- H --

|  |  |
| --- | --- |
| haar2level | Multi Level Decomposition of unidimensional data |
| hclust2phylog | Create phylogeny |
| hdpg | Genetic Variation In Human Populations |
| housetasks | Contingency Table |
| humDNAm | human mitochondrial DNA restriction data |

## -- I --

|  |  |
| --- | --- |
| ichtyo | Point sampling of fish community |
| inertia.dudi | Statistics of inertia in a one-table analysis |
| irishdata | Geary's Irish Data |
| is.dudi | Duality Diagram |
| is.euclid | Is a Distance Matrix Euclidean ? |
| is.ktab | the class of objects 'ktab' (K-tables) |

## -- J --

|  |  |
| --- | --- |
| julliot | Seed dispersal |
| jv73 | K-tables Multi-Regions |

## -- K --

|  |  |
| --- | --- |
| kcponds | Ponds in a nature reserve |
| kdist | the class of objects 'kdist' (K distance matrices) |
| kdist.cor | Mixed-variables coefficient of distance |
| kdist2ktab | Transformation of K distance matrices (object 'kdist') into K Euclidean representations (object 'ktab') |
| kdisteuclid | a way to obtain Euclidean distance matrices |
| kplot | Generic Function for Multiple Graphs in a K-tables Analysis |
| kplot.foucart | Multiple Graphs for the Foucart's Correspondence Analysis |
| kplot.mcoa | Multiple Graphs for a Multiple Co-inertia Analysis |
| kplot.mfa | Multiple Graphs for a Multiple Factorial Analysis |
| kplot.pta | Multiple Graphs for a Partial Triadic Analysis |
| kplot.sepan | Multiple Graphs for Separated Analyses in a K-tables |
| kplot.sepan.coa | Multiple Graphs for Separated Analyses in a K-tables |
| kplot.statis | Multiple Graphs of a STATIS Analysis |
| kplotX.mdpcoa | Multiple Double Principal Coordinate Analysis |
| krandtest | Class of the Permutation Tests (in C). |
| ktab | the class of objects 'ktab' (K-tables) |
| ktab.data.frame | Creation of K-tables from a data frame |
| ktab.list.df | Creating a K-tables from a list of data frames. |
| ktab.list.dudi | Creation of a K-tables from a list of duality diagrams |
| ktab.match2ktabs | STATIS and Co-Inertia : Analysis of a series of paired ecological tables |
| ktab.util.addfactor<- | the class of objects 'ktab' (K-tables) |
| ktab.util.names | the class of objects 'ktab' (K-tables) |
| ktab.within | Process to go from a Within Analysis to a K-tables |

## -- L --

|  |  |
| --- | --- |
| lascaux | Genetic/Environment and types of variables |
| ldist.ktab | Mixed-variables coefficient of distance |
| lingoes | Transformation of a Distance Matrix for becoming Euclidean |
| lizards | Phylogeny and quantitative traits of lizards |

## -- M --

|  |  |
| --- | --- |
| macaca | Landmarks |
| macon | Wine Tasting |
| macroloire | Assemblages of Macroinvertebrates in the Loire River (France) |
| mafragh | Phyto-Ecological Survey |
| mantel.randtest | Mantel test (correlation between two distance matrices (in C).) |
| mantel.rtest | Mantel test (correlation between two distance matrices (in R).) |
| mantelkdist | Tests of randomization between distances applied to 'kdist' objetcs |
| maples | Phylogeny and quantitative traits of flowers |
| mariages | Correspondence Analysis Table |
| mcoa | Multiple CO-inertia Analysis |
| mdpcoa | Multiple Double Principal Coordinate Analysis |
| meau | Ecological Data : sites-variables, sites-species, where and when |
| meaudret | Ecological Data : sites-variables, sites-species, where and when |
| mfa | Multiple Factorial Analysis |
| microsatt | Genetic Relationships between cattle breeds with microsatellites |
| mjrochet | Phylogeny and quantitative traits of teleos fishes |
| mld | Multi Level Decomposition of unidimensional data |
| mollusc | Faunistic Communities and Sampling Experiment |
| monde84 | Global State of the World in 1984 |
| morphosport | Athletes' Morphology |
| mstree | Minimal Spanning Tree |
| multispati | Multivariate spatial analysis |
| multispati.randtest | Multivariate spatial autocorrelation test (in C) |
| multispati.rtest | Multivariate spatial autocorrelation test |

## -- N --

|  |  |
| --- | --- |
| nb2neig | Neighbourhood Graphs |
| neig | Neighbourhood Graphs |
| neig.util.GtoL | Neighbourhood Graphs |
| neig.util.LtoG | Neighbourhood Graphs |
| neig2mat | Neighbourhood Graphs |
| neig2nb | Neighbourhood Graphs |
| newick.eg | Phylogenetic trees in Newick format |
| newick2phylog | Create phylogeny |
| newick2phylog.addtools | Create phylogeny |
| niche | Method to Analyse a pair of tables : Environmental and Faunistic Data |
| niche.param | Method to Analyse a pair of tables : Environmental and Faunistic Data |
| nipals | Non-linear Iterative Partial Least Squares (NIPALS) algorithm |
| njplot | Phylogeny and trait of bacteria |

## -- O --

|  |  |
| --- | --- |
| olympic | Olympic Decathlon |
| optimEH | Nee and May's optimizing process |
| oribatid | Oribatid mite |
| originality | Originality of a species |
| orisaved | Maximal or minimal amount of originality saved under optimal conditions |
| orthobasis | Orthonormal basis for orthonormal transform |
| orthobasis.circ | Orthonormal basis for orthonormal transform |
| orthobasis.haar | Orthonormal basis for orthonormal transform |
| orthobasis.line | Orthonormal basis for orthonormal transform |
| orthobasis.listw | Orthonormal basis for orthonormal transform |
| orthobasis.mat | Orthonormal basis for orthonormal transform |
| orthobasis.neig | Orthonormal basis for orthonormal transform |
| orthogram | Orthonormal decomposition of variance |
| ours | A table of Qualitative Variables |

## -- P --

|  |  |
| --- | --- |
| palm | Phylogenetic and quantitative traits of amazonian palm trees |
| pap | Taxonomy and quantitative traits of carnivora |
| pcaiv | Principal component analysis with respect to instrumental variables |
| pcaivortho | Principal Component Analysis with respect to orthogonal instrumental variables |
| pcoscaled | Simplified Analysis in Principal Coordinates |
| perthi02 | Contingency Table with a partition in Molecular Biology |
| phylog | Phylogeny |
| phylog.extract | Phylogeny |
| phylog.permut | Phylogeny |
| PI2newick | Import data files from Phylogenetic Independance Package |
| plot.4thcorner | Functions to compute the fourth-corner statistic |
| plot.betcoi | Between-class coinertia analysis |
| plot.between | Between-Class Analysis |
| plot.coinertia | Coinertia Analysis |
| plot.corkdist | Tests of randomization between distances applied to 'kdist' objetcs |
| plot.discrimin | Linear Discriminant Analysis (descriptive statistic) |
| plot.dpcoa | Double principal coordinate analysis |
| plot.foucart | K-tables Correspondence Analysis with the same rows and the same columns |
| plot.krandtest | Class of the Permutation Tests (in C). |
| plot.mcoa | Multiple CO-inertia Analysis |
| plot.mfa | Multiple Factorial Analysis |
| plot.multispati | Multivariate spatial analysis |
| plot.niche | Method to Analyse a pair of tables : Environmental and Faunistic Data |
| plot.pcaiv | Principal component analysis with respect to instrumental variables |
| plot.phylog | Plot phylogenies |
| plot.procuste | Simple Procruste Rotation between two sets of points |
| plot.pta | Partial Triadic Analysis of a K-tables |
| plot.randtest | Class of the Permutation Tests (in C). |
| plot.rlq | RLQ analysis |
| plot.rtest | Class of the Permutation Tests (in R). |
| plot.sepan | Separated Analyses in a K-tables |
| plot.statis | STATIS, a method for analysing K-tables |
| plot.witcoi | Within-class coinertia analysis |
| plot.within | Within Analyses |
| poly2area | Graphical Display of Areas |
| prep.binary | Mixed-variables coefficient of distance |
| prep.circular | Mixed-variables coefficient of distance |
| prep.fuzzy | Mixed-variables coefficient of distance |
| prep.fuzzy.var | Fuzzy Correspondence Analysis and Fuzzy Principal Components Analysis |
| prep.mdpcoa | Multiple Double Principal Coordinate Analysis |
| presid2002 | Results of the French presidential elections of 2002 |
| print.4thcorner | Functions to compute the fourth-corner statistic |
| print.amova | Analysis of molecular variance |
| print.apqe | Apportionment of Quadratic Entropy |
| print.betcoi | Between-class coinertia analysis |
| print.between | Between-Class Analysis |
| print.coinertia | Coinertia Analysis |
| print.corkdist | Tests of randomization between distances applied to 'kdist' objetcs |
| print.discrimin | Linear Discriminant Analysis (descriptive statistic) |
| print.dpcoa | Double principal coordinate analysis |
| print.dudi | Duality Diagram |
| print.foucart | K-tables Correspondence Analysis with the same rows and the same columns |
| print.kdist | the class of objects 'kdist' (K distance matrices) |
| print.krandtest | Class of the Permutation Tests (in C). |
| print.ktab | the class of objects 'ktab' (K-tables) |
| print.mcoa | Multiple CO-inertia Analysis |
| print.mfa | Multiple Factorial Analysis |
| print.multispati | Multivariate spatial analysis |
| print.neig | Neighbourhood Graphs |
| print.niche | Method to Analyse a pair of tables : Environmental and Faunistic Data |
| print.nipals | Non-linear Iterative Partial Least Squares (NIPALS) algorithm |
| print.orthobasis | Orthonormal basis for orthonormal transform |
| print.pcaiv | Principal component analysis with respect to instrumental variables |
| print.phylog | Phylogeny |
| print.procuste | Simple Procruste Rotation between two sets of points |
| print.pta | Partial Triadic Analysis of a K-tables |
| print.randtest | Class of the Permutation Tests (in C). |
| print.rlq | RLQ analysis |
| print.rtest | Class of the Permutation Tests (in R). |
| print.sepan | Separated Analyses in a K-tables |
| print.statis | STATIS, a method for analysing K-tables |
| print.witcoi | Within-class coinertia analysis |
| print.within | Within Analyses |
| procella | Phylogeny and quantitative traits of birds |
| procuste | Simple Procruste Rotation between two sets of points |
| procuste.randtest | Monte-Carlo Test on the sum of the singular values of a procustean rotation (in C). |
| procuste.rtest | Monte-Carlo Test on the sum of the singular values of a procustean rotation (in R). |
| pta | Partial Triadic Analysis of a K-tables |

## -- Q --

|  |  |
| --- | --- |
| quasieuclid | Transformation of a distance matrice to a Euclidean one |

## -- R --

|  |  |
| --- | --- |
| radial.phylog | Plot phylogenies |
| randEH | Nee and May's random process |
| randtest | Class of the Permutation Tests (in C). |
| randtest.amova | Permutation tests on an analysis of molecular variance (in C). |
| randtest.between | Monte-Carlo Test on the between-groups inertia percentage (in C). |
| randtest.cca | Monte-Carlo Test on the percentage of explained (i.e. constrained) inertia |
| randtest.coinertia | Monte-Carlo test on a Co-inertia analysis (in C). |
| randtest.discrimin | Monte-Carlo Test on a Discriminant Analysis (in C). |
| randtest.pcaiv | Monte-Carlo Test on the percentage of explained (i.e. constrained) inertia |
| randtest.pcaivortho | Monte-Carlo Test on the percentage of explained (i.e. constrained) inertia |
| randtest.rlq | RLQ analysis |
| rankrock | Ordination Table |
| reciprocal.coa | Reciprocal scaling after a correspondence analysis |
| reconst | Reconstitution of Data from a Duality Diagram |
| reconst.coa | Reconstitution of Data from a Duality Diagram |
| reconst.pca | Reconstitution of Data from a Duality Diagram |
| redo.dudi | Duality Diagram |
| rhizobium | Genetic structure of two nitrogen fixing bacteria influenced by geographical isolation and host specialization |
| rhone | Physico-Chemistry Data |
| rlq | RLQ analysis |
| row.names.ktab | the class of objects 'ktab' (K-tables) |
| row.names<-.ktab | the class of objects 'ktab' (K-tables) |
| rpjdl | Avifauna and Vegetation |
| rtest | Class of the Permutation Tests (in R). |
| rtest.between | Monte-Carlo Test on the between-groups inertia percentage (in R). |
| rtest.discrimin | Monte-Carlo Test on a Discriminant Analysis (in R). |
| rtest.niche | Method to Analyse a pair of tables : Environmental and Faunistic Data |
| Rtoade4 | Format Change Utility |
| RV.rtest | Monte-Carlo Test on the sum of eigenvalues of a co-inertia analysis (in R). |
| RVdist.randtest | Tests of randomization on the correlation between two distance matrices (in R). |
| RVkdist | Tests of randomization between distances applied to 'kdist' objetcs |

## -- S --

|  |  |
| --- | --- |
| s.arrow | Plot of the factorial maps for the projection of a vector basis |
| s.chull | Plot of the factorial maps with polygons of contour by level of a factor |
| s.class | Plot of factorial maps with representation of point classes |
| s.corcircle | Plot of the factorial maps of a correlation circle |
| s.distri | Plot of a frequency distribution |
| s.hist | Display of a scatterplot and its two marginal histograms |
| s.image | Graph of a variable using image and contour |
| s.kde2d | Scatter Plot with Kernel Density Estimate |
| s.label | Scatter Plot |
| s.logo | Representation of an object in a graph by a picture |
| s.match | Plot of Paired Coordinates |
| s.match.class | Scatterplot of two sets of coordinates and a partionning into classes |
| s.multinom | Graph of frequency profiles (useful for instance in genetic) |
| s.traject | Trajectory Plot |
| s.value | Representation of a value in a graph |
| santacatalina | Indirect Ordination |
| sarcelles | Array of Recapture of Rings |
| scalewt | Centring and Scaling a Matrix of Any Weighting |
| scatter | Scatter Plot |
| scatter.acm | Plot of the factorial maps in a Multiple Correspondence Analysis |
| scatter.coa | Plot of the factorial maps for a correspondence analysis |
| scatter.dudi | Plot of the Factorial Maps |
| scatter.fca | Plot of the factorial maps for a fuzzy correspondence analysis |
| scatter.nipals | Non-linear Iterative Partial Least Squares (NIPALS) algorithm |
| scatter.pco | Principal Coordinates Analysis |
| scatterutil.base | Graphical utility functions |
| scatterutil.chull | Graphical utility functions |
| scatterutil.convrot90 | Graphical utility functions |
| scatterutil.eigen | Graphical utility functions |
| scatterutil.ellipse | Graphical utility functions |
| scatterutil.eti | Graphical utility functions |
| scatterutil.eti.circ | Graphical utility functions |
| scatterutil.grid | Graphical utility functions |
| scatterutil.legend.bw.square | Graphical utility functions |
| scatterutil.legend.square.grey | Graphical utility functions |
| scatterutil.legendgris | Graphical utility functions |
| scatterutil.logo | Representation of an object in a graph by a picture |
| scatterutil.scaling | Graphical utility functions |
| scatterutil.sco | Graphical utility functions |
| scatterutil.star | Graphical utility functions |
| scatterutil.sub | Graphical utility functions |
| sco.boxplot | Representation of the link between a variable and a set of qualitative variables |
| sco.class | 1D plot of a numeric score and a factor with labels |
| sco.distri | Representation by mean- standard deviation of a set of weight distributions on a numeric score |
| sco.gauss | Relationships between one score and qualitative variables |
| sco.label | 1D plot of a numeric score with labels |
| sco.match | 1D plot of a pair of numeric scores with labels |
| sco.quant | Graph to Analyse the Relation between a Score and Quantitative Variables |
| score | Graphs for One Dimension |
| score.acm | Graphs to study one factor in a Multiple Correspondence Analysis |
| score.coa | Reciprocal scaling after a correspondence analysis |
| score.mix | Graphs to Analyse a factor in a Mixed Analysis |
| score.pca | Graphs to Analyse a factor in PCA |
| scores.neig | Neighbourhood Graphs |
| scoreutil.base | Graphs for One Dimension |
| seconde | Students and Subjects |
| sepan | Separated Analyses in a K-tables |
| skulls | Morphometric Evolution |
| statico | STATIS and Co-Inertia : Analysis of a series of paired ecological tables |
| statis | STATIS, a method for analysing K-tables |
| steppe | Transect in the Vegetation |
| summary.4thcorner | Functions to compute the fourth-corner statistic |
| summary.coinertia | Coinertia Analysis |
| summary.corkdist | Tests of randomization between distances applied to 'kdist' objetcs |
| summary.dist | Is a Distance Matrix Euclidean ? |
| summary.mcoa | Multiple CO-inertia Analysis |
| summary.mfa | Multiple Factorial Analysis |
| summary.multispati | Multivariate spatial analysis |
| summary.neig | Neighbourhood Graphs |
| summary.rlq | RLQ analysis |
| summary.sepan | Separated Analyses in a K-tables |
| summary.witwit | Internal Correspondence Analysis |
| supcol | Projections of Supplementary Columns |
| supcol.coa | Projections of Supplementary Columns |
| supcol.default | Projections of Supplementary Columns |
| suprow | Projections of Supplementary Rows |
| suprow.coa | Projections of Supplementary Rows |
| suprow.default | Projections of Supplementary Rows |
| suprow.pca | Projections of Supplementary Rows |
| symbols.phylog | Representation of a quantitative variable in front of a phylogenetic tree |
| syndicats | Two Questions asked on a Sample of 1000 Respondents |

## -- T --

|  |  |
| --- | --- |
| t.dudi | Duality Diagram |
| t.ktab | the class of objects 'ktab' (K-tables) |
| t3012 | Average temperatures of 30 French cities |
| tab.names | the class of objects 'ktab' (K-tables) |
| tab.names.ktab | the class of objects 'ktab' (K-tables) |
| tab.names<- | the class of objects 'ktab' (K-tables) |
| tab.names<-.ktab | the class of objects 'ktab' (K-tables) |
| table.cont | Plot of Contingency Tables |
| table.dist | Graph Display for Distance Matrices |
| table.paint | Plot of the arrays by grey levels |
| table.phylog | Plot arrays in front of a phylogenetic tree |
| table.prepare | Plot of the Arrays |
| table.value | Plot of the Arrays |
| tarentaise | Mountain Avifauna |
| taxo.eg | Examples of taxonomy |
| taxo2phylog | Create phylogeny |
| testdim | Function to perform a test of dimensionality |
| testdim.pca | Function to perform a test of dimensionality |
| tintoodiel | Tinto and Odiel estuary geochemistry |
| tithonia | Phylogeny and quantitative traits of flowers |
| tortues | Morphological Study of the Painted Turtle |
| toxicity | Homogeneous Table |
| triangle.biplot | Triangular Plotting |
| triangle.class | Triangular Representation and Groups of points |
| triangle.param | Triangular Plotting |
| triangle.plot | Triangular Plotting |
| triangle.posipoint | Triangular Plotting |
| trichometeo | Pair of Ecological Data |

## -- U --

|  |  |
| --- | --- |
| ungulates | Phylogeny and quantitative traits of ungulates. |
| uniquewt.df | Elimination of Duplicated Rows in a Array |

## -- V --

|  |  |
| --- | --- |
| variance.phylog | The phylogenetic ANOVA |
| vegtf | Vegetation in Trois-Fontaines |
| veuvage | Example for Centring in PCA |

## -- W --

|  |  |
| --- | --- |
| westafrica | Freshwater fish zoogeography in west Africa |
| within | Within Analyses |
| withincoinertia | Within-class coinertia analysis |
| withinpca | Normed within Principal Component Analysis |
| witwit.coa | Internal Correspondence Analysis |
| witwitsepan | Internal Correspondence Analysis |
| woangers | Plant assemblages in woodlands of the conurbation of Angers (France) |
| worksurv | French Worker Survey (1970) |

## -- Y --

|  |  |
| --- | --- |
| yanomama | Distance Matrices |

## -- Z --

|  |  |
| --- | --- |
| zealand | Road distances in New-Zealand |

## -- misc --

|  |  |
| --- | --- |
| [.kdist | the class of objects 'kdist' (K distance matrices) |
| [.ktab | the class of objects 'ktab' (K-tables) |
