# Supplementary material for: DockAnalyse: an application for the analysis of protein-protein interactions
Source: BMC Struct Biol. 2010 Oct 22;10:37. doi: 10.1186/1472-6807-10-37 (PMC2987812; doi:10.1186/1472-6807-10-37)
Supplement: Additional file 1 — DockAnalyse. 4 R program files, 4 Perl scripts, 2 text files and 3 zippped libraries. [file 1472-6807-10-37-S1.ZIP › clusterSim_0.37-1.zip!/clusterSim/html/00Index.html]

R: Searching for optimal clustering procedure for a data set

# Searching for optimal clustering procedure for a data set


---

## Documentation for package ‘clusterSim’ version 0.37-1

- DESCRIPTION file.

## Help Pages

|  |  |
| --- | --- |
| cluster.Description | Descriptive statistics calculated separately for each cluster and variable |
| cluster.Gen | Random cluster generation with known structure of clusters |
| cluster.Sim | Determination of optimal clustering procedure for a data set |
| data.Normalization | Types of variable normalization formulas |
| data\_binary | Binary data |
| data\_interval | Interval data |
| data\_mixed | Mixed data |
| data\_nominal | Nominal data |
| data\_ordinal | Ordinal data |
| data\_ratio | Ratio data |
| data\_symbolic | Symbolic interval data |
| dist.BC | Calculates Bray-Curtis distance measure for ratio data |
| dist.GDM | Calculates Generalized Distance Measure |
| dist.SM | Calculates Sokal-Michener distance measure for nominal variables |
| dist.Symbolic | Calculates distance between symbolic interval-valued data |
| GDM | Calculates Generalized Distance Measure |
| GDM1 | Calculates Generalized Distance Measure |
| GDM2 | Calculates Generalized Distance Measure |
| HINoV.Mod | Modification of Carmone, Kara & Maxwell Heuristic Identification of Noisy Variables (HINoV) method |
| HINoV.Symbolic | Modification of Carmone, Kara & Maxwell Heuristic Identification of Noisy Variables (HINoV) method for symbolic interval data |
| index.DB | Calculates Davies-Bouldin's index |
| index.G1 | Calculates Calinski-Harabasz pseudo F-statistic |
| index.G2 | Calculates G2 internal cluster quality index |
| index.G3 | Calculates G3 internal cluster quality index |
| index.Gap | Calculates Tibshirani, Walther and Hastie gap index |
| index.H | Calculates Hartigan index |
| index.KL | Calculates Krzanowski-Lai index |
| index.S | Calculates Rousseeuw's Silhouette internal cluster quality index |
| initial.Centers | Calculation of initial clusters centers for k-means like alghoritms |
| plotCategorial | Plot categorial data on a scatterplot matrix |
| plotCategorial3d | Plot categorial data with three-dimensional plots |
| plotInterval | Plot symbolic interval-valued data on a scatterplot matrix |
| replication.Mod | Modification of replication analysis for cluster validation |
