# Supplementary material for: DockAnalyse: an application for the analysis of protein-protein interactions
Source: BMC Struct Biol. 2010 Oct 22;10:37. doi: 10.1186/1472-6807-10-37 (PMC2987812; doi:10.1186/1472-6807-10-37)
Supplement: Additional file 1 — DockAnalyse. 4 R program files, 4 Perl scripts, 2 text files and 3 zippped libraries. [file 1472-6807-10-37-S1.ZIP › clusterSim_0.37-1.zip!/clusterSim/pdf/clusterGen_details.pdf]

Random cluster generation with known structure of clusters

Models

Metric data **(`dataType="m"`)**

`model=1`. No cluster structure. The observations are simulated from the uniform distribution over the unit hypercube.

`model=2`. The observations are independently drawn from normal distribution with means and covariances are taken from arguments `means` and `cov`.

`model=3`. Two elongated clusters in 2 dimensions. The observations in each of two clusters are independent bivariate normal random variables with means (0, 0), (1, 5), and covariance matrix Σ ($\sigma_{jj}=1$, $\sigma_{jl}=-0.9$).

`model=4`. Three elongated clusters in 2 dimensions. The observations are independently drawn from bivariate normal distribution with means (0, 0), (1.5, 7), (3, 14) and covariance matrix Σ ($\sigma_{jj}=1$, $\sigma_{jl}=-0.9$).

`model=5`. Three elongated clusters in 3 dimensions. The observations are independently drawn from multivariate normal distribution with means (1.5, 6, –3), (3, 12, –6), (4.5, 18, –9), and identity covariance matrix Σ, where $\sigma_{jj}=1$ $(1\le j\le 3)$, $\sigma_{12}=\sigma_{13}=-0.9$, and $\sigma_{23}=0.9$.

`model=6`. Five clusters in 2 dimensions that are not well separated. The observations are independently drawn from bivariate normal distribution with means (5, 5), (–3, 3), (3, –3), (0, 0), (–5, –5), and identity covariance matrix Σ ($\sigma_{jj}=1$, $\sigma_{jl}=0.9$).

`model=7`. Five clusters in 3 dimensions that are not well separated. The observations are independently drawn from multivariate normal distribution with means (5, 5, 5), (–3, 3, –3), (3, –3, 3), (0, 0, 0), (–5, –5, –5), and covariance matrix Σ, where $\sigma_{jj}=1$ $(1\le j\le 3)$, and $\sigma_{jl}=0.9$ $(1\le j\ne l\le 3)$.

`model=8`. Five clusters in 2 dimensions. The observations are independently drawn from bivariate normal distribution with means (0, 0), (0, 10), (5, 5), (10, 0), (10, 10), and identity covariance matrix Σ ($\sigma_{jj}=1$, $\sigma_{jl}=0$).

`model=9`. Five clusters in 3 dimensions. The observations are independently drawn from multivariate normal distribution with means (0, 0, 0), (10, 10, 10), (–10, –10, –10), (10, –10, 10), (–10, 10, 10), and identity covariance matrix Σ, where $\sigma_{jj}=3$ $(1\le j\le 3)$, and $\sigma_{jl}=2$ $(1\le j\ne l\le 3)$.

`model=10`. Four clusters in 2 dimensions. The observations are independently drawn from bivariate normal distribution with means (–4, 5), (5, 14), (14, 5), (5, –4), and identity covariance matrix Σ ($\sigma_{jj}=1$, $\sigma_{jl}=0$).

`model=11`. Four clusters in 3 dimensions. The observations are independently drawn from multivariate normal distribution with means (–4, 5, –4), (5, 14, 5), (14, 5, 14), (5, –4, 5), and identity covariance matrix Σ, where $\sigma_{jj}=1$ $(1\le j\le 3)$, and $\sigma_{jl}=0$ $(1\le j\ne l\le 3)$.

`model=12`. Four clusters in 1 dimension. The observations are independently drawn from univariate normal distribution with means –2, 4, 10, 16 respectively, and identity variance $\sigma_j^2=0.5$ $(1\le j\le 4)$.

`model=13`. Three elongated clusters in 2 dimensions. The observations are independently drawn from bivariate normal distribution with means (0, 0), (1.5, 7), (3, 14) and covariance matrices

$$\Sigma_1=\begin{bmatrix}1 & -0.9\\ -0.9 & 1\end{bmatrix},\ \Sigma_2=\begin{bmatrix}1.5 & 0\\ 0 & 1.5\end{bmatrix},\ \Sigma_3=\begin{bmatrix}1 & 0.5\\ 0.5 & 1\end{bmatrix}.$$

`model=14`. Four clusters in 3 dimensions. The observations are independently drawn from multivariate normal distribution with means (–4, 5, –4), (5, 14, 5), (14, 5, 14), (5, –4, 5), and covariance matrices $\Sigma_1=\begin{bmatrix}1&0&0\\0&1&0\\0&0&1\end{bmatrix}$, $\Sigma_2=\begin{bmatrix}1&-0.9&-0.9\\-0.9&1&0.9\\-0.9&0.9&1\end{bmatrix}$, $\Sigma_3=\begin{bmatrix}1&0.9&0.9\\0.9&1&0.9\\0.9&0.9&1\end{bmatrix}$, $\Sigma_4=\begin{bmatrix}3&2&2\\2&3&2\\2&2&3\end{bmatrix}$.

`model=15`. Five clusters in 3 dimensions that are not well separated. The observations are independently drawn from multivariate normal distribution with means (5, 5, 5), (–3, 3, –3), (3, –3, 3), (0, 0, 0), (–5, –5, –5), and covariance matrices $\Sigma_1=\begin{bmatrix}1&-0.9&-0.9\\-0.9&1&0.9\\-0.9&0.9&1\end{bmatrix}$, $\Sigma_2=\begin{bmatrix}0.5&0&0\\0&1&0\\0&0&2\end{bmatrix}$, $\Sigma_3=\begin{bmatrix}1&0.9&0.9\\0.9&1&0.9\\0.9&0.9&1\end{bmatrix}$, $\Sigma_4=\begin{bmatrix}1&0.6&0.6\\0.6&1&0.6\\0.6&0.6&1\end{bmatrix}$, $\Sigma_5=\begin{bmatrix}1&0&0\\0&1&0\\0&0&1\end{bmatrix}$.

`model=16`. Two elongated clusters in 2 dimensions. The observations in each of two clusters are independent bivariate normal random variables with means (0, 0), (1, 5), and covariance matrices $\Sigma_1=\begin{bmatrix}1&-0.9\\-0.9&1\end{bmatrix}$, $\Sigma_2=\begin{bmatrix}1&0.5\\0.5&1\end{bmatrix}$.

`model=21,22,...` – if `fixedCov=TRUE` means should be read from *means_<modelNumber>.csv* and covariance matrix for all clusters should be read from *cov_<modelNumber>.csv* and if `fixedCov=FALSE` means should be read from *means_<modelNumber>.csv* and covariance matrices should be read separately for each cluster from *cov_<modelNumber>_<clusterNumber>.csv*, e.g. (`inputType="csv"`)

means_21.csv	*cov_21_1.csv*	*cov_21_2.csv*
`"V1","V2"`<br>`"1",4,8`<br>`"2",0,4`	`"V1","V2"`<br>`"1",1.0,0.9`<br>`"2",0.9,1.0`	`"V1","V2"`<br>`"1",1.0,-0.9`<br>`"2",-0.9,1.0`

***Ordinal data* (`dataType="o"`).** The clusters in models 1, 2, ... contain continuous data and a discretization process is performed on each variable to obtain ordinal data. The number of categories k_j determines the width of each class intervals: $\left[\max_i\{x_{ij}\}-\min_i\{x_{ij}\}\right]/k_j$. Independently for each variable each class interval receive category $1,\ldots,k_j$ and the actual value of variable x_{ij} is replaced by these categories.

***Symbolic interval data* (`dataType="s"`).** To obtain symbolic interval data the data were generated for each model twice into sets *A* and *B* and minimal (maximal) value of $\left\{x_{ij}^A, x_{ij}^B\right\}$ is treated as the beginning (the end) of an interval.

Noisy variables. The noisy variables are simulated independently from the uniform distribution. We require that the variations of noisy variables in the generated data are similar to non-noisy variables (see Milligan [1985], Qiu and Joe [2006], p. 322).

Outliers (for metric and symbolic interval data only)**.** The outliers are generated independently for each variable for the whole data set from uniform distribution (the default range is [1, 10]). The generated values are randomly added to maximum of *j*-th variable or subtracted from minimum of *j*-th variable.

References

Qiu, W., Joe, H. (2006), *Generation of random clusters with specified degree of separation*, "Journal of Classification", vol. 23, 315-334.

Steinley, D., Henson, R. (2005), *OCLUS: an analytic method for generating clusters with known overlap*, "Journal of Classification", vol. 22, 221-250.

Walesiak, M., Dudek, A. (2007), *Identification of noisy variables for nonmetric and symbolic data in cluster analysis*, In: Data Analysis, Machine Learning, and Applications, Springer-Verlag, Berlin, Heidelberg (in press).
