# Supplementary material for: DockAnalyse: an application for the analysis of protein-protein interactions
Source: BMC Struct Biol. 2010 Oct 22;10:37. doi: 10.1186/1472-6807-10-37 (PMC2987812; doi:10.1186/1472-6807-10-37)
Supplement: Additional file 1 — DockAnalyse. 4 R program files, 4 Perl scripts, 2 text files and 3 zippped libraries. [file 1472-6807-10-37-S1.ZIP › clusterSim_0.37-1.zip!/clusterSim/pdf/clusterSim_details.pdf]

`cluster.Sim` (clusterSim)

Paths characteristics in determination of optimal clustering procedure for a data set

No.	Steps in a typical cluster analysis	Path's number									
		1		2	3	4	5	6	7	8	9
I	Selection of objects and variables	data matrix $[x_{ij}]$									
II	Measurement scale of variables	ratio	ratio	interval or mixed[1]	ordinal[2]	multi-state nominal[3]	binary	ratio	interval or mixed[1]	ratio	interval or mixed[1]
	Selection of normalization formula[4]	`n6` – `n11`	`n1` – `n5`	`n1` – `n5`	N.A.	N.A.		without normalization		`n6-n11` / `n1-n5`	`n1-n5`
	Transformed measurement scale of variables	ratio	interval	interval	ordinal	multi-state nominal	binary	ratio	interval or mixed[1]	ratio / interval	interval
III	Selection of distance measure[5]	`d1` – `d7`	`d1` – `d5`	`d1` – `d5`	`d8`	`d9`	`b1` – `b10`	`d1` – `d7`	`d1` – `d5`	N.A.	
IV	Selection of clustering method[6]	`m1` – `m8`								`m9`	
V	Maximal number of possible variants	[(6 x 7 x 5)+ (6 x 1 x 3)] + [(5 x 5 x 5) +(5 x 1 x 3)] = 368		(5 x 5 x 5) + (5 x 1 x 3) = 140	1 x 5 = 5	1 x 5 = 5	10 x 5 = 50	(7 x 5) + (1 x 3) = 38	(5 x 5) + (1 x 3) = 28	11	5
	Number of all classifications	`LK` = (`maxClusterNo` − `minClusterNo` + 1) · LW_p, where<br>`minClusterNo` minimal number of clusters,<br>`maxClusterNo` maximal number of clusters,<br>LW_p – number of variants for *p*-th path.									
	Internal cluster quality index	1. Calinski & Harabasz (`G1`)[7]<br>2. Baker & Hubert (`G2`)<br>3. Hubert & Levine (`G3`)<br>4. Silhouette (`S`)<br>5. Krzanowski & Lai (`KL`)[7]			1. N.A.<br>2. `G2`<br>3. `G3`<br>4. `S`<br>5. N.A.			1. `G1`<br>2. `G2`<br>3. `G3`<br>4. `S`<br>5. `KL`		1. `G1`<br>2. N.A.<br>3. N.A.<br>4. N.A.<br>5. `KL`	

[1] Ratio & interval.

[2] We can use ratio, interval or mixed data (ratio, interval, ordinal), however these data are treated as ordinal because in the construction of the GDM2 distance measure only such relations as: "equal to", "higher than", "lower than" are taken into account.

[3] We can use ratio, interval, ordinal or mixed data (ratio, interval, ordinal, nominal), however these data are treated as nominal because in the construction of the Sokal & Michener distance measure only such relations as: "equal to", "not equal to" are taken into account.

[4] `n1` – (x-mean)/sd, `n2` – (x-Me)/MAD, `n3` – (x-mean)/range, `n4` – (x-min)/range, `n5` – (x-mean)/max[abs(x-mean)], `n6` – (x/sd), `n7` – (x/range), `n8` – (x/max), `n9` – (x/mean), `n10` – (x/sum), `n11` – x/sqrt(SSQ).

[5] `d1` – Manhattan, `d2` – Euclidean, `d3` – Chebychev (max), `d4` – squared Euclidean, `d5` – GDM1, `d6` – Canberra, `d7` – Bray-Curtis; `d8` – GDM2, `d9` – Sokal & Michener; `b1` – `b10` (available in R `dist.binary` procedure): `b1` = Jaccard; `b2` = Sokal & Michener; `b3` = Sokal & Sneath (1); `b4` = Rogers & Tanimoto; `b5` = Czekanowski; `b6` = Gower & Legendre (1); `b7` = Ochiai; `b8` = Sokal & Sneath (2); `b9` = Phi of Pearson; `b10` = Gower & Legendre (2).

[6] `m1` – single link, `m2` – complete link, `m3` – average link, `m4` – McQuitty, `m5` – *k*-medoids (PAM), `m6` – Ward, `m7` – centroid, `m8` – median, `m9` – *k*-means. For clustering methods `m6` – `m8` squared Euclidean distance is used only.

[7] with argument `centrotypes="centroids"`.

N.A. – Not Applicable.

Source: Walesiak, M., Dudek, A. (2006), *Symulacyjna optymalizacja wyboru procedury klasyfikacyjnej dla danego typu danych – oprogramowanie komputerowe i wyniki badan*, Prace Naukowe AE we Wroclawiu no. 1126, 120-129.
