# Supplementary material for: DockAnalyse: an application for the analysis of protein-protein interactions
Source: BMC Struct Biol. 2010 Oct 22;10:37. doi: 10.1186/1472-6807-10-37 (PMC2987812; doi:10.1186/1472-6807-10-37)
Supplement: Additional file 1 — DockAnalyse. 4 R program files, 4 Perl scripts, 2 text files and 3 zippped libraries. [file 1472-6807-10-37-S1.ZIP › clusterSim_0.37-1.zip!/clusterSim/pdf/dataNormalization_details.pdf]

data.Normalization(clusterSim)

Types of variable normalization formulas

1	Selection of objects and variables	data matrix $[x_{ij}]$		
2	Variable scale level	ratio	ratio	interval
	Selection of variable normalization formula	n6 – quotient transformation (x/sd)<br>n7 – quotient transformation (x/range)<br>n8 – quotient transformation (x/max)<br>n9 – quotient transformation (x/mean)<br>n10 – quotient transformation (x/sum)<br>n11 – quotient transformation x/sqrt(SSQ)	n1 – standardization<br>n2 – Weber standardization<br>n3 – unitization<br>n4 – unitization with zero minimum<br>n5 – normalization in range [–1, 1]	n1 – standardization<br>n2 – Weber standardization<br>n3 – unitization<br>n4 – unitization with zero minimum<br>n5 – normalization in range [–1 ,1]
	Transformed variable scale level	ratio	interval	interval

(n1) $$z_{ij} = s_j^{-1} x_{ij} - \bar{x}_j s_j^{-1},$$

(n2) $$z_{ij} = (x_{ij} - Me_j) \big/ 1,4826 \cdot MAD_j ,$$

(n3) $$z_{ij} = r_j^{-1} x_{ij} - \bar{x}_j r_j^{-1},$$

(n4) $$z_{ij} = \left[x_{ij} - \min_i \{x_{ij}\} \right] \Big/ r_j$$

(n5) $$z_{ij} = (x_{ij} - \bar{x}_j) \big/ \max_i \left| x_{ij} - \bar{x}_j \right|$$

(n6) $$x_{ij} / s_j$$

(n7) $$x_{ij} / r_j$$

n(8) $$x_{ij} \big/ \max_i \{x_{ij}\}$$

(n9) $$x_{ij} / \bar{x}_j$$

(n10) $$x_{ij} \Big/ \sum_{i=1}^{n} x_{ij}$$

(n11) $$x_{ij} \Big/ \sqrt{\sum_{i=1}^{n} x_{ij}^2}$$

where: $x_{ij}(z_{ij})$ – i-th observation on j-th variable (i-th normalized observation on j-th variable),

$\bar{x}_j, s_j$ – mean and standard deviation for j-th variable,

Me_j, MAD_j – median and median absolute deviation for j-th variable,

$r_j = \max_i \{x_{ij}\} - \min_i \{x_{ij}\}$.

References

Gatnar, E., Walesiak, M. (Eds.) (2004), *Metody statystycznej analizy wielowymiarowej w badaniach marketingowych [Multivariate statistical analysis methods in marketing research]*, Wydawnictwo AE, Wroclaw, 35-38.

Jajuga, K., Walesiak, M. (2000), *Standardisation of data set under different measurement scales*, In: R. Decker, W. Gaul (Eds.), *Classification and information processing at the turn of the millennium*, Springer-Verlag, Berlin, Heidelberg, 105-112.

Milligan, G.W., Cooper, M.C. (1988), *A study of standardization of variables in cluster analysis*, “Journal of Classification”, vol. 5, 181-204.
