# Supplementary material for: DockAnalyse: an application for the analysis of protein-protein interactions
Source: BMC Struct Biol. 2010 Oct 22;10:37. doi: 10.1186/1472-6807-10-37 (PMC2987812; doi:10.1186/1472-6807-10-37)
Supplement: Additional file 1 — DockAnalyse. 4 R program files, 4 Perl scripts, 2 text files and 3 zippped libraries. [file 1472-6807-10-37-S1.ZIP › clusterSim_0.37-1.zip!/clusterSim/pdf/distBC_details.pdf]

dist.BC(clusterSim)

Bray-Curtis distance measure d_{ik} for ratio data

$$d_{ik} = \frac{\sum_{j=1}^{m} \left| x_{ij} - x_{kj} \right|}{\sum_{j=1}^{m} (x_{ij} + x_{kj})},$$

where: $i,k = 1,\ldots,n$ – the number of object,

$j = 1,\ldots,m$ – the number of variable,

x_{ij} (x_{kj}) – *i*-th (*k*-th) observation on *j*-th variable.

References

Cormack, R.M. (1971), *A review of classification (with discussion)*, "Journal of the Royal Statistical Society", ser. A, part 3, 321-367.
