# Supplementary material for: DockAnalyse: an application for the analysis of protein-protein interactions
Source: BMC Struct Biol. 2010 Oct 22;10:37. doi: 10.1186/1472-6807-10-37 (PMC2987812; doi:10.1186/1472-6807-10-37)
Supplement: Additional file 1 — DockAnalyse. 4 R program files, 4 Perl scripts, 2 text files and 3 zippped libraries. [file 1472-6807-10-37-S1.ZIP › clusterSim_0.37-1.zip!/clusterSim/pdf/distGDM_details.pdf]

dist.GDM(clusterSim)

The General Distance Measure

(see Walesiak [2006])

The General Distance Measure (*GDM*) is given by the following equation:

$$GDM = \frac{1}{2} - \frac{\sum_{j=1}^{m} w_j a_{ikj} b_{kij} + \sum_{j=1}^{m} \sum_{\substack{l=1 \\ l \neq i,k}}^{n} w_j a_{ilj} b_{klj}}{2\left[\sum_{j=1}^{m} \sum_{l=1}^{n} w_j a_{ilj}^2 \cdot \sum_{j=1}^{m} \sum_{l=1}^{n} w_j b_{klj}^2\right]^{\frac{1}{2}}}, \qquad (1)$$

where: $i,k,l = 1,\dots,n$ – the number of object,

$j = 1,\dots,m$ – the number of variable,

x_{ij} (x_{kj}, x_{lj}) – *i*-th (*k*-th, *l*-th) observation on *j*-th variable,

w_j – weight of *j*-th variable: $w_j \in [0;1]$ and $\sum_{j=1}^{m} w_j = 1$ or $w_j \in [0;m]$ and $\sum_{j=1}^{m} w_j = m$.

For the variables measured on **ratio and (or) interval scale** we take a_{ipj}, b_{krj} given as (Walesiak [2002]):

$$\begin{aligned} a_{ipj} &= x_{ij} - x_{pj} \quad \text{for} \quad p = k,l \\ b_{krj} &= x_{kj} - x_{rj} \quad \text{for} \quad r = i,l \end{aligned}, \qquad (2)$$

$$GDM1 = \frac{1}{2} - \frac{\sum_{j=1}^{m} w_j (x_{ij} - x_{kj})(x_{kj} - x_{ij}) + \sum_{j=1}^{m} \sum_{\substack{l=1 \\ l \neq i,k}}^{n} w_j (x_{ij} - x_{lj})(x_{kj} - x_{lj})}{2\left[\sum_{j=1}^{m} \sum_{l=1}^{n} w_j (x_{ij} - x_{lj})^2 \cdot \sum_{j=1}^{m} \sum_{l=1}^{n} w_j (x_{kj} - x_{lj})^2\right]^{\frac{1}{2}}}. \qquad (3)$$

For the variables measured on **ordinal scale** we take a_{ipj}, b_{krj} given as (Walesiak [1993], pp. 44-45):

$$a_{ipj}\left(b_{krj}\right) = \begin{cases} 1 & \text{if} \quad x_{ij} > x_{pj} \left(x_{kj} > x_{rj}\right) \\ 0 & \text{if} \quad x_{ij} = x_{pj} \left(x_{kj} = x_{rj}\right), \\ -1 & \text{if} \quad x_{ij} < x_{pj} \left(x_{kj} < x_{rj}\right) \end{cases} \quad \text{for} \quad p = k,l\,;\ l,r = i,l\,, \qquad (4)$$

$$GDM2 = \frac{1}{2} - \frac{\sum_{j=1}^{m} w_j a_{ikj} b_{kij} + \sum_{j=1}^{m} \sum_{\substack{l=1 \\ l \neq i,k}}^{n} w_j a_{ilj} b_{klj}}{2\left[\sum_{j=1}^{m} \sum_{l=1}^{n} w_j a_{ilj}^2 \cdot \sum_{j=1}^{m} \sum_{l=1}^{n} w_j b_{klj}^2\right]^{\frac{1}{2}}}. \qquad (5)$$

The properties of the General Distance Measure

– it can be applied when the variables are measured on the ordinal (*GDM*2), interval and ratio scale (*GDM*1),

– it takes values from the [0; 1] interval. Value 0 indicates that for the compared objects *i*, *k* between corresponding observations of variables, only relations "equal to" take place. For *GDM*2 the value 1 indicates that for the compared objects *i*, *k* between corresponding observations on ordinal

variables, relations “greater than” take place or relations “greater than” and relations “equal to”, if they are held for other objects (i.e. objects numbered $l = 1, \ldots, n$; where $l \neq i, k$),

– it satisfies the conditions: non-negative, reflexive, and symmetric (for all $i, k = 1, \ldots, n$),

– the empirical analysis proves that distance GDM sometimes does not satisfy the triangle inequality,

– it needs at least one pair of non-identical objects in order to avoid zero in the denominator,

– the transformation of data by any strictly increasing function (*GDM*2) or by any linear function (*GDM*1) does not change the value of distance.

References

Jajuga, K., Walesiak, M., Bak, A. (2003), *On the general distance measure*, In: M. Schwaiger, O. Opitz (Eds.), *Exploratory data analysis in empirical research*, Springer-Verlag, Berlin, Heidelberg, 104-109.

Walesiak, M. (1993), *Statystyczna analiza wielowymiarowa w badaniach marketingowych* [*Multivariate Statistical Analysis in Marketing Research*], Wroclaw University of Economics, Research Papers no. 654.

Walesiak, M. (1999), *Distance Measure for Ordinal Data*, Argumenta Oeconomica, No. 2 (8), 167-173.

Walesiak, M. (2002), *Propozycja uogolnionej miary odleglosci w statystycznej analizie wielowymiarowej*, In: J. Paradysz (Ed.), *Statystyka regionalna w słuzbie samorzadu lokalnego i biznesu*, Internetowa Oficyna Wydawnicza, Centrum Statystyki Regionalnej, AE w Poznaniu, Poznan, 115-121.

Walesiak, M. (2006), *Uogólniona miara odległości w statystycznej analizie wielowymiarowej* [*The Generalized Distance Measure in multivariate statistical analysis]*, Wydanie drugie rozszerzone, Wydawnictwo AE, Wroclaw.

Walesiak, M. (2008), *Cluster analysis with clusterSim computer program and R environment*, Acta Universitatis Lodziensis. Folia Oeconomica, No. 216, 303-311.

Walesiak, M., Dziechciarz, J., Bąk, A. (1998), *Ordinal variables in the segmentation of advertisement receivers*, In: A. Rizzi, N. Vichi, H.H. Bock (Eds.), *Advances in Data Science and Classification*, Springer, Heidelberg, 655-662.
