# Supplementary material for: DockAnalyse: an application for the analysis of protein-protein interactions
Source: BMC Struct Biol. 2010 Oct 22;10:37. doi: 10.1186/1472-6807-10-37 (PMC2987812; doi:10.1186/1472-6807-10-37)
Supplement: Additional file 1 — DockAnalyse. 4 R program files, 4 Perl scripts, 2 text files and 3 zippped libraries. [file 1472-6807-10-37-S1.ZIP › clusterSim_0.37-1.zip!/clusterSim/pdf/distSM_details.pdf]

dist.SM(clusterSim)

Sokal-Michener distance measure d_{ik} for nominal data

$$d_{ik} = \frac{\sum_{j=1}^{m}(1 - g_{ik}^{(j)})}{m} = \frac{m - m_r}{m},$$

where: $i, k = 1, \ldots, n$ – the number of object,

$j = 1, \ldots, m$ – the number of variable,

x_{ij} (x_{kj}) – *i*-th (*k*-th) observation on *j*-th variable,

$$g_{ik}^{(j)} = \begin{cases} 1, & x_{ij} = x_{kj} \\ 0, & x_{ij} \neq x_{kj} \end{cases},$$

m_r – number of variables where $x_{ij} = x_{kj}$ (for *i*-th and *k*-th object).

References

Gatnar, E., Walesiak, M. (Eds.) (2004), *Metody statystycznej analizy wielowymiarowej w badaniach marketingowych [Multivariate statistical analysis methods in marketing research]*, Wydawnictwo AE, Wroclaw, p. 43.

Kaufman, L., Rousseeuw, P.J. (1990), *Finding groups in data: an introduction to cluster analysis*, Wiley, New York, p. 28.
