# Supplementary material for: DockAnalyse: an application for the analysis of protein-protein interactions
Source: BMC Struct Biol. 2010 Oct 22;10:37. doi: 10.1186/1472-6807-10-37 (PMC2987812; doi:10.1186/1472-6807-10-37)
Supplement: Additional file 1 — DockAnalyse. 4 R program files, 4 Perl scripts, 2 text files and 3 zippped libraries. [file 1472-6807-10-37-S1.ZIP › clusterSim_0.37-1.zip!/clusterSim/pdf/distSymbolic_details.pdf]

dist.Symbolic(clusterSim)

Ichino and Yaguchi dissimilarity measure for variables

$$\phi(A_j, B_j) = |A_j \oplus B_j| - |A_j \otimes B_j| + \gamma\left(2 \cdot |A_j \otimes B_j| - |A_j| - |B_j|\right),$$

where: $\phi(A_j, B_j)$ – dissimilarity measure of symbolic variables,

A_j, B_j – symbolic variables of any type,

$\oplus$ – Cartesian join,

$\otimes$ – Cartesian meet,

$|\ |$ – means the length of an interval for continuous data or the number of elements of an set of values,

γ – parameter <0, ½ >.

Ichino and Yaguchi measure for objects (U_2)

$$d_q(a_1, b_1) = \left(\sqrt[q]{\sum_{j=1}^{p} \varphi(A_j, B_j)^q} \right),$$

where: $d_q(a_1, b_1)$ – Ichino and Yaguchi measure (sometimes called extended Minkowski metric – see Diday [2000]),

q – integer number greater or equal 1,

$a_1 = (A_1, A_2, \ldots, A_p)$, $b_1 = (B_1, B_2, \ldots, B_p)$ – two symbolic objects containing p symbolic variables,

$\varphi(A_j, B_j)$ – Ichino and Yaguchi dissimilarity measure for variables.

Hausdorff distance measure (H)

$$\max\left\{ \max_{\alpha \in [\underline{A}, \overline{A}]} \left\{ \min_{\beta \in [\underline{B}, \overline{B}]} d(\alpha, \beta) \right\}, \max_{\beta \in [\underline{B}, \overline{B}]} \left\{ \min_{\alpha \in [\underline{A}, \overline{A}]} d(\alpha, \beta) \right\} \right\},$$

where: $H(a_1, b_1)$ – Hausdorff distance,

$a_1 = (A_1, A_2, \ldots, A_p)$, $b_1 = (B_1, B_2, \ldots, B_p)$ – two symbolic objects containing p symbolic interval-valued variables. Each variable A_i is an interval in a form $[\underline{A_i}, \overline{A_i}]$,

$d(\ldots)$ – Euclidean distance.

References

Billard, L., Diday, E. (2006), *Symbolic data analysis. Conceptual statistics and data mining*, Wiley, Chichester.

Bock H.H., Diday E. (Eds.) (2000), *Analysis of symbolic data. Explanatory methods for extracting statistical information from complex data*, Springer Verlag, Berlin.
