# Supplementary material for: DockAnalyse: an application for the analysis of protein-protein interactions
Source: BMC Struct Biol. 2010 Oct 22;10:37. doi: 10.1186/1472-6807-10-37 (PMC2987812; doi:10.1186/1472-6807-10-37)
Supplement: Additional file 1 — DockAnalyse. 4 R program files, 4 Perl scripts, 2 text files and 3 zippped libraries. [file 1472-6807-10-37-S1.ZIP › clusterSim_0.37-1.zip!/clusterSim/pdf/HINoVMod_details.pdf]

HINoV.Mod(clusterSim)

Modification of Carmone, Kara & Maxwell Heuristic Identification of Noisy Variables (HINoV)

Algorithm for metric data (see Carmone, Kara and Maxwell [1999])

Step 1. Data matrix containing m normalized variables measured on metric scale (ratio, interval) and n objects ($i = 1,\ldots,n;\ j = 1,\ldots,m$) is a starting point.

Step 2. Cluster, via `kmeans` method, the observed data separately for each j-th variable for a given number of cluster u. It is possible to use clustering methods based on distance matrix (`pam` or any hierarchical agglomerative method: `single`, `complete`, `average`, `mcquitty`, `median`, `centroid`, `Ward`).

Step 3. Calculate adjusted Rand indices R_{jl} ($j,l = 1,\ldots,m$) for partitions formed from all distinct pairs of the m variables ($j \neq l$). Due to fact that adjusted Rand (Rand) index is symmetrical we need to calculate $m(m-1)/2$ values.

Step 4. Construct $m \times m$ adjusted Rand matrix (`parim`). Sum rows (or columns) for each j-th variable $R_{j\bullet} = \sum_{l=1}^{m} R_{jl}$ (`topri`):

parim topri

$$\begin{bmatrix} M_1 \\ M_2 \\ \vdots \\ M_j \\ \vdots \\ M_m \end{bmatrix} \begin{bmatrix} & R_{12} & \cdots & R_{1l} & \cdots & R_{1m} \\ R_{21} & & \cdots & R_{2l} & \cdots & R_{2m} \\ \vdots & \vdots & \vdots & \vdots & \vdots & \vdots \\ R_{j1} & R_{j2} & \cdots & R_{jl} & \cdots & R_{jm} \\ \vdots & \vdots & \vdots & \vdots & \vdots & \vdots \\ R_{m1} & R_{m2} & \cdots & R_{ml} & \cdots & \end{bmatrix} \begin{bmatrix} R_{1\bullet} \\ R_{2\bullet} \\ \vdots \\ R_{j\bullet} \\ \vdots \\ R_{m\bullet} \end{bmatrix}$$

Step 5. Rank `topri` values $R_{1\bullet}, R_{2\bullet}, \ldots, R_{m\bullet}$ in decreasing order (`stopri`) and plot the scree diagram. The size of the `topri` values indicate the contribution of that variable to the cluster structure. A scree diagram identifies sharp changes in `topri` values. Relatively low-valued `topri` variables (the noisy variables) are identified and eliminated from further analysis (say h variables).

Step 6. Run cluster analysis (based on the same classification method) with the selected $m-h$ variables.

Modification of Carmone, Kara & Maxwell Heuristic Identification of Noisy Variables (HINoV) method for nonmetric data[1] differs in steps 1, 2, and 6 (see Walesiak [2007]):

Step 1. Data matrix $[x_{ij}]$ containing m ordinal and/or nominal variables and n objects is a starting point.

Step 2. For each j-th variable we receive natural clusters, where number of clusters equals number of categories for that variable (for instance five for Likert scale or seven for semantic differential scale).

[1] For nonmetric variables (ordinal, nominal) contain not to many categories (for nonmetric variables where number of objects is much more than number of categories).

Step 6. Run cluster analysis with one of clustering methods based on distance appropriate to non-metric data (GDM2 for ordinal data – see Jajuga, Walesiak & Bak [2003]; Sokal and Michener distance for nominal data) with the selected $m-h$ variables.

References

Carmone F.J., Kara A., Maxwell S. (1999), *HINoV: a new method to improve market segment definition by identifying noisy variables*, "Journal of Marketing Research", November, vol. 36, 501-509.

Hubert L.J., Arabie P. (1985), *Comparing partitions*, "Journal of Classification", no. 1, 193-218.

Jajuga, K., Walesiak, M., Bąk, A. (2003): *On the General Distance Measure*, In: M. Schwaiger, and O. Opitz (Eds.), *Exploratory Data Analysis in Empirical Research*, Springer-Verlag, Berlin, Heidelberg, 104-109.

Rand W.M. (1971), *Objective criteria for the evaluation of clustering methods*, "Journal of the American Statistical Association", no. 336, 846-850.

Walesiak M. (2005), *Variable selection for cluster analysis – approaches, problems, methods,* Plenary Session of the Committee on Statistics and Econometrics of the Polish Academy of Sciences, 15 March, Wroclaw.
