# Supplementary material for: DockAnalyse: an application for the analysis of protein-protein interactions
Source: BMC Struct Biol. 2010 Oct 22;10:37. doi: 10.1186/1472-6807-10-37 (PMC2987812; doi:10.1186/1472-6807-10-37)
Supplement: Additional file 1 — DockAnalyse. 4 R program files, 4 Perl scripts, 2 text files and 3 zippped libraries. [file 1472-6807-10-37-S1.ZIP › clusterSim_0.37-1.zip!/clusterSim/pdf/HINoVSymbolic_details.pdf]

Modification of Carmone, Kara & Maxwell Heuristic Identification of Noisy Variables (HINoV) for symbolic interval data

Algorithm of HINoV method for symbolic interval data (see Walesiak and Dudek [2007])

Step 1. Symbolic data array containing *m* symbolic interval variables and *n* objects is a starting point.

Step 2. Cluster the observed data with one of clustering methods (`pam`, `single`, `complete`, `average`, `mcquitty`, `median`, `centroid`, `Ward`) based on distance appropriate to symbolic interval data (e.g. Hausdorff distance) separately for each *j*-th variable for a given number of cluster *u*.

Step 3. Calculate adjusted Rand indices R_{jl} $(j,l=1,\dots,m)$ for partitions formed from all distinct pairs of the *m* variables ($j \neq l$). Due to fact that adjusted Rand (Rand) index is symmetrical we need to calculate $m(m-1)/2$ values.

Step 4. Construct $m \times m$ adjusted Rand matrix (`parim`). Sum rows (or columns) for each *j*-th variable $R_{j\bullet} = \sum_{l=1}^{m} R_{jl}$ (`topri`):

$$\begin{bmatrix} M_1 \\ M_2 \\ \vdots \\ M_j \\ \vdots \\ M_m \end{bmatrix} \overset{\text{parim}}{\begin{bmatrix} & R_{12} & \dots & R_{1l} & \dots & R_{1m} \\ R_{21} & & \dots & R_{2l} & \dots & R_{2m} \\ \vdots & \vdots & \vdots & \vdots & \vdots & \vdots \\ R_{j1} & R_{j2} & \dots & R_{jl} & \dots & R_{jm} \\ \vdots & \vdots & \vdots & \vdots & \vdots & \vdots \\ R_{m1} & R_{m2} & \dots & R_{ml} & \dots & \end{bmatrix}} \overset{\text{topri}}{\begin{bmatrix} R_{1\bullet} \\ R_{2\bullet} \\ \vdots \\ R_{j\bullet} \\ \vdots \\ R_{m\bullet} \end{bmatrix}}$$

Step 5. Rank `topri` values $R_{1\bullet}, R_{2\bullet}, \dots, R_{m\bullet}$ in decreasing order (`stopri`) and plot the scree diagram. The size of the `topri` values indicate the contribution of that variable to the cluster structure. A scree diagram identifies sharp changes in `topri` values. Relatively low-valued `topri` variables (the noisy variables) are identified and eliminated from further analysis (say *h* variables).

Step 6. Run cluster analysis (based on the same classification method) with the selected $m-h$ variables.

References

Carmone F.J., Kara A., Maxwell S. (1999), *HINoV: a new method to improve market segment definition by identifying noisy variables*, “Journal of Marketing Research”, November, vol. 36, 501-509.

Hubert L.J., Arabie P. (1985), *Comparing partitions*, “Journal of Classification”, no. 1, 193-218.

Rand W.M. (1971), *Objective criteria for the evaluation of clustering methods*, “Journal of the American Statistical Association”, no. 336, 846-850.

Walesiak M., Dudek A. (2007), *Identification of noisy variables for nonmetric and symbolic data in cluster analysis*, 31st Annual Conference of the German Classification Society (GfKl): *Data Analysis, Machine Learning, and Applications* (Freiburg, March, 7-9).
