# Supplementary material for: DockAnalyse: an application for the analysis of protein-protein interactions
Source: BMC Struct Biol. 2010 Oct 22;10:37. doi: 10.1186/1472-6807-10-37 (PMC2987812; doi:10.1186/1472-6807-10-37)
Supplement: Additional file 1 — DockAnalyse. 4 R program files, 4 Perl scripts, 2 text files and 3 zippped libraries. [file 1472-6807-10-37-S1.ZIP › clusterSim_0.37-1.zip!/clusterSim/pdf/indexDB_details.pdf]

index.DB(clusterSim)

Davies-Bouldin's index

$$DB(u)=\frac{1}{u}\sum_{r=1}^{u}\max_{\substack{s\\ r\neq s}}\left(\frac{S_r+S_s}{d_{rs}}\right),$$

where: $r,s=1,\ldots,u$ – cluster number,

u – number of clusters ($u\geq 2$),

$i,k=1,\ldots,n$ – object number,

n – number of objects,

P_r, P_s – r-th, s-th cluster,

$\mathbf{z}_r=(z_{r1},\ldots,z_{rm})$ – centroid or medoid of cluster P_r,

$j=1,\ldots,m$ – variable number,

$d_{rs}=\sqrt[p]{\sum_{j=1}^{m}\left|z_{rj}-z_{sj}\right|^p}$ – distance between centroids or medoids of clusters P_r and P_s (for $p=1$ Manhattan distance, for $p=2$ Euclidean distance),

$S_r=\sqrt[q]{\frac{1}{n_r}\sum_{i\in P_r}\sum_{j=1}^{m}\left|x_{ij}^{r}-z_{rj}\right|^q}$ – dispersion measure of a cluster P_r (for $q=1$ the average distance of objects in cluster P_r to the centroid or medoid of cluster P_r; for $q=2$ the standard deviation of the distance of objects in cluster P_r to the centroid or medoid of cluster P_r),

n_r (n_s) – number of objects in cluster P_r (P_s).

The value of u, which minimizes $DB(u)$, is regarded as specifying the number of clusters.

The Davies-Bouldin's index have two limitations:

– index is not permitted if the number of clusters equals the number of objects ($DB(u)=0$ if $u=n$),

– the certain number of clusters to have only one object is allowed. An unlimited number of single member clusters is not permitted.

$DB(u)$ is a very good cluster separation measure if each cluster contain at least two objects.

References

Davies, D.L., Bouldin, D.W. (1979), *A cluster separation measure*, "IEEE Transactions on Pattern Analysis and Machine Intelligence", vol. 1, no. 2, 224-227.
