# Supplementary material for: DockAnalyse: an application for the analysis of protein-protein interactions
Source: BMC Struct Biol. 2010 Oct 22;10:37. doi: 10.1186/1472-6807-10-37 (PMC2987812; doi:10.1186/1472-6807-10-37)
Supplement: Additional file 1 — DockAnalyse. 4 R program files, 4 Perl scripts, 2 text files and 3 zippped libraries. [file 1472-6807-10-37-S1.ZIP › clusterSim_0.37-1.zip!/clusterSim/pdf/indexG1_details.pdf]

index.G1(clusterSim)

**Caliński-Harabasz pseudo F-Statistic**

$$G1(u) = \frac{\text{trace}(\mathbf{B}_u)/(u-1)}{\text{trace}(\mathbf{W}_u)/(n-u)},$$

where: $\mathbf{X} = \{x_{ij}\}$, $i = 1,\ldots,n$; $j = 1,\ldots,m$ – data matrix,

$n$ – number of objects,

$m$ – number of variables,

$u$ – number of clusters ($u$ = 2,…, $n-1$),

$\mathbf{W}_u = \sum_r \sum_{i \in C_r} (\mathbf{x}_{ri} - \overline{\mathbf{x}}_r)(\mathbf{x}_{ri} - \overline{\mathbf{x}}_r)^T$ – within-group dispersion matrix for data clustered into $u$ clusters,

$\mathbf{B}_u = \sum_r n_r (\overline{\mathbf{x}}_r - \overline{\mathbf{x}})(\overline{\mathbf{x}}_r - \overline{\mathbf{x}})^T$ – between-group dispersion matrix for data clustered into $u$ clusters,

$r = 1,\ldots,u$ – cluster number,

$\overline{\mathbf{x}}_r$ – centroid or medoid of cluster $r$,

$\overline{\mathbf{x}}$ – centroid or medoid of data matrix,

$C_r$ – the indices of objects in cluster $r$,

$n_r$ – number of objects in cluster $r$.

The value of $u$, which maximizes $G1(u)$, is regarded as specifying the number of clusters.

**References**

Caliński, R.B., Harabasz, J. (1974), *A dendrite method for cluster analysis*, „Communications in Statistics", vol. 3, 1-27.

Everitt, B.S., Landau, E., Leese, M. (2001), *Cluster analysis*, Arnold, London, p. 103.

Gatnar, E., Walesiak, M. (Eds.) (2004), *Metody statystycznej analizy wielowymiarowej w badaniach marketingowych [Multivariate statistical analysis methods in marketing research]*, Wydawnictwo AE, Wroclaw, p. 338.

Gordon, A.D. (1999), *Classification*, Chapman & Hall/CRC, London, p. 62.

Milligan, G.W., Cooper, M.C. (1985), *An examination of procedures of determining the number of cluster in a data set*, "Psychometrika", vol. 50, no. 2, 159-179.
