# Supplementary material for: DockAnalyse: an application for the analysis of protein-protein interactions
Source: BMC Struct Biol. 2010 Oct 22;10:37. doi: 10.1186/1472-6807-10-37 (PMC2987812; doi:10.1186/1472-6807-10-37)
Supplement: Additional file 1 — DockAnalyse. 4 R program files, 4 Perl scripts, 2 text files and 3 zippped libraries. [file 1472-6807-10-37-S1.ZIP › clusterSim_0.37-1.zip!/clusterSim/pdf/indexG2_details.pdf]

index.G2(clusterSim)

G2 internal cluster quality index
– Baker & Hubert adaptation of Goodman & Kruskal's Gamma statistics

$$G2(u) = \frac{s(+) - s(-)}{s(+) + s(-)},$$
$$G2(u) \in [-1,\ 1],$$

where: $s(+)$ – number of concordant comparisons (within-cluster dissimilarity is strictly less than a between-cluster dissimilarity),

$s(-)$ – number of discordant comparisons (within-cluster dissimilarity is strictly greater than a between-cluster dissimilarity),

u – number of clusters ($u = 2, \ldots, n-1$),

n – number of objects.

The value of u, which maximizes $G2(u)$, is regarded as specifying the number of clusters.

References

Everitt, B.S., Landau, E., Leese, M. (2001), *Cluster analysis*, Arnold, London, p. 104.

Gatnar, E., Walesiak, M. (Eds.) (2004), *Metody statystycznej analizy wielowymiarowej w badaniach marketingowych [Multivariate statistical analysis methods in marketing research]*, Wydawnictwo AE, Wroclaw, p. 339.

Gordon, A.D. (1999), *Classification*, Chapman & Hall/CRC, London, p. 62.

Hubert, L. (1974), *Approximate evaluation technique for the single-link and complete-link hierarchical clustering procedures*, "Journal of the American Statistical Association", vol. 69, no. 347, 698-704.

Milligan, G.W., Cooper, M.C. (1985), *An examination of procedures of determining the number of cluster in a data set*, "Psychometrika", vol. 50, no. 2, 159-179.
