# Supplementary material for: DockAnalyse: an application for the analysis of protein-protein interactions
Source: BMC Struct Biol. 2010 Oct 22;10:37. doi: 10.1186/1472-6807-10-37 (PMC2987812; doi:10.1186/1472-6807-10-37)
Supplement: Additional file 1 — DockAnalyse. 4 R program files, 4 Perl scripts, 2 text files and 3 zippped libraries. [file 1472-6807-10-37-S1.ZIP › clusterSim_0.37-1.zip!/clusterSim/pdf/indexG3_details.pdf]

index.G3(clusterSim)

## G3 Hubert & Levine internal cluster quality index

$$G3(u) = \frac{D(u) - r \cdot D_{\min}}{r \cdot D_{\max} - r \cdot D_{\min}}, \; D_{\min} \neq D_{\max},$$

$$G3(u) \in (0, \; 1),$$

where: $D(u)$ – all within-cluster dissimilarities,

$r$ – number of within-cluster dissimilarities,

$D_{\min}$ – smallest within-cluster dissimilarity,

$D_{\max}$ – largest within-cluster dissimilarity,

$u$ – number of clusters ($u = 2, \ldots, n-2$).

The value of $u$, which minimizes $G3(u)$, is regarded as specifying the number of clusters.

**References**


Gatnar, E., Walesiak, M. (Eds.) (2004), *Metody statystycznej analizy wielowymiarowej w badaniach marketingowych [Multivariate statistical analysis methods in marketing research]*, Wydawnictwo AE, Wroclaw, p. 339.

Gordon, A.D. (1999), *Classification*, Chapman & Hall/CRC, London, p. 62.

Milligan, G.W., Cooper, M.C. (1985), *An examination of procedures of determining the number of cluster in a data set*, “Psychometrika”, vol. 50, no. 2, 159-179.
