# Supplementary material for: DockAnalyse: an application for the analysis of protein-protein interactions
Source: BMC Struct Biol. 2010 Oct 22;10:37. doi: 10.1186/1472-6807-10-37 (PMC2987812; doi:10.1186/1472-6807-10-37)
Supplement: Additional file 1 — DockAnalyse. 4 R program files, 4 Perl scripts, 2 text files and 3 zippped libraries. [file 1472-6807-10-37-S1.ZIP › clusterSim_0.37-1.zip!/clusterSim/pdf/IndexGap_details.pdf]

index.Gap(clusterSim)

Tibshirani, Walther and Hastie gap index

Step 1. Cluster the observed data $\mathbf{X} = \{x_{ij}\}$, $i = 1,\ldots,n$; $j = 1,\ldots,m$ (via e.g. any hierarchical clustering method, pam, *k*-means), varying the total number of clusters from $u = 1,\ldots,n$, giving within-dispersion measures:

$$W_u = \text{trace}(\mathbf{W}_u),$$

where: $\mathbf{W}_u = \sum_r \sum_{i \in C_r} (\mathbf{x}_{ri} - \bar{\mathbf{x}}_r)(\mathbf{x}_{ri} - \bar{\mathbf{x}}_r)^T$ – within-group dispersion matrix for data clustered into u clusters,

$\mathbf{x}_{ri}$ – *m*-dimensional vector of observations of the *i*-th object in cluster *r*,

$\bar{\mathbf{x}}_r$ – centroid or medoid of cluster r,

$r = 1,\ldots,u$ – cluster number,

u – number of clusters ($u = 1,\ldots,n$),

n – number of objects,

m – number of variables,

C_r – the indices of objects in cluster r.

Step 2. Generate *B* reference data sets, using the uniform prescription:

a) generate each reference variable uniformly over the range of the observed values for that variable,

or

b) generate the reference variables from a uniform distribution over a box aligned with the principal components of the data. In detail, if $\mathbf{X} = \{x_{ij}\}$ is our $n \times m$ data matrix, assume that the columns have mean 0 and compute the singular value decomposition $\mathbf{X} = \mathbf{UDV}^T$. We transform via $\mathbf{X}' = \mathbf{XV}$ and then draw uniform features $\mathbf{Z}'$ over the ranges of the columns of $\mathbf{X}'$, as in method a) above. Finally we back-transform via $\mathbf{Z} = \mathbf{Z}'\mathbf{V}^T$ to give reference data $\mathbf{Z}$,

and cluster each one (using the same clustering method) giving within-dispersion measures W_{ub} ($b = 1,\ldots,B$; $u = 1,\ldots,n-1$). Compute the (estimated) gap statistic:

$$\text{Gap}(u) = \frac{1}{B}\sum_{b=1}^{B} log\, W_{ub} - log\, W_u$$

Step 3. Compute the standard deviation of $\{ log\, W_{ub} \}$, $b = 1,\ldots,B$:

$$sd_u = \sqrt{\frac{1}{B}\sum_{b=1}^{B}\left(log\, W_{ub} - \bar{l}\right)^2},$$

where: $\bar{l} = \frac{1}{B}\sum_{b=1}^{B} log\, W_{ub}$,

and define

$$s_u = sd_u \sqrt{1 + 1/B}$$

Step 4. Finally choose the number of clusters via finding the smallest u such that:

$$\text{Gap}(u) \geq \text{Gap}(u+1) - s_{u+1} \quad (u = 1,\ldots,n-2)$$

References

Tibshirani R., Walther G., Hastie T. (2001), *Estimating the number of clusters in a data set via the gap statistic*, „Journal of the Royal Statistical Society”, ser. B, vol. 63, part 2, 411-423.
