# Supplementary material for: DockAnalyse: an application for the analysis of protein-protein interactions
Source: BMC Struct Biol. 2010 Oct 22;10:37. doi: 10.1186/1472-6807-10-37 (PMC2987812; doi:10.1186/1472-6807-10-37)
Supplement: Additional file 1 — DockAnalyse. 4 R program files, 4 Perl scripts, 2 text files and 3 zippped libraries. [file 1472-6807-10-37-S1.ZIP › clusterSim_0.37-1.zip!/clusterSim/pdf/indexH_details.pdf]

index.H(clusterSim)

Hartigan index

$$H(u)=\left(\frac{tr\mathbf{W}_u}{tr\mathbf{W}_{u+1}}-1\right)(n-u-1),$$

where: $\mathbf{X}=\{x_{ij}\}$, $i=1,\ldots,n$; $j=1,\ldots,m$ – data matrix,

n – number of objects,

m – number of variables,

$\mathbf{W}_u=\sum_r\sum_{i\in C_r}(\mathbf{x}_{ri}-\bar{\mathbf{x}}_r)(\mathbf{x}_{ri}-\bar{\mathbf{x}}_r)^T$ – within-group dispersion matrix for data clustered into u clusters,

$\mathbf{x}_{ri}$ – m-dimensional vector of observations of the i-th object in cluster r,

$\bar{\mathbf{x}}_r$ – centroid or medoid of cluster r,

$r=1,\ldots,u$ – cluster number,

u – number of clusters ($u=1,\ldots,n-2$),

C_r – the indices of objects in cluster r.

The estimated number of clusters is the smallest $u\geq 1$ such that $H(u)\leq 10$.

References

Hartigan, J. (1975), *Clustering algorithms*, Wiley, New York.

Milligan, G.W., Cooper, M.C. (1985), *An examination of procedures of determining the number of cluster in a data set*, “Psychometrika”, vol. 50, no. 2, 159-179.

Tibshirani R., Walther G., Hastie T. (2001), *Estimating the number of clusters in a data set via the gap statistic*, „Journal of the Royal Statistical Society”, ser. B, vol. 63, part 2, 411-423.
