# Supplementary material for: DockAnalyse: an application for the analysis of protein-protein interactions
Source: BMC Struct Biol. 2010 Oct 22;10:37. doi: 10.1186/1472-6807-10-37 (PMC2987812; doi:10.1186/1472-6807-10-37)
Supplement: Additional file 1 — DockAnalyse. 4 R program files, 4 Perl scripts, 2 text files and 3 zippped libraries. [file 1472-6807-10-37-S1.ZIP › clusterSim_0.37-1.zip!/clusterSim/pdf/indexKL_details.pdf]

index.KL(clusterSim)

Krzanowski and Lai index

$$KL(u) = \left|\frac{DIFF_u}{DIFF_{u+1}}\right|,$$

$$DIFF_u = (u-1)^{2/m} tr\mathbf{W}_{u-1} - u^{2/m} tr\mathbf{W}_u,$$

where: $\mathbf{X} = \{x_{ij}\}$, $i = 1,\ldots,n$; $j = 1,\ldots,m$ – data matrix,

n – number of objects,

m – number of variables,

$\mathbf{W}_u = \sum_r \sum_{i \in C_r} (\mathbf{x}_{ri} - \bar{\mathbf{x}}_r)(\mathbf{x}_{ri} - \bar{\mathbf{x}}_r)^T$ – within-group dispersion matrix for data clustered into u clusters,

$\mathbf{x}_{ri}$ – m-dimensional vector of observations of the i-th object in cluster r,

$\bar{\mathbf{x}}_r$ – centroid or medoid of cluster r,

$r = 1,\ldots,u$ – cluster number,

u – number of clusters ($u = 2,\ldots,n-2$),

C_r – the indices of objects in cluster r.

The value of u, which maximizes $KL(u)$, is regarded as specifying the number of clusters.

References

Krzanowski , W.J., Lai, Y.T. (1988), *A criterion for determining the number of groups in a data set using sum of squares clustering*, “Biometrics”, 44, no. 1, 23-34.

Tibshirani R., Walther G., Hastie T. (2001), *Estimating the number of clusters in a data set via the gap statistic*, „Journal of the Royal Statistical Society”, ser. B, vol. 63, part 2, 411-423.
