# Supplementary material for: DockAnalyse: an application for the analysis of protein-protein interactions
Source: BMC Struct Biol. 2010 Oct 22;10:37. doi: 10.1186/1472-6807-10-37 (PMC2987812; doi:10.1186/1472-6807-10-37)
Supplement: Additional file 1 — DockAnalyse. 4 R program files, 4 Perl scripts, 2 text files and 3 zippped libraries. [file 1472-6807-10-37-S1.ZIP › clusterSim_0.37-1.zip!/clusterSim/pdf/indexS_details.pdf]

index.S(clusterSim)

Rousseeuw Silhouette internal cluster quality index

$$S(u) = \sum_{i=1}^{n} S(i) / n$$

$$S(u) \in [-1,\ 1],$$

where: $S(i) = \dfrac{b(i) - a(i)}{\max\{a(i); b(i)\}}$,

$i, k = 1, \ldots, n$ – number of object,

P_r, P_s – *r*-th, *s*-th cluster,

u – number of clusters,

n_r, n_s – number of objects in cluster P_r, P_s,

$a(i) = \sum_{k \in \{P_r \setminus i\}} d_{ik} \Big/ (n_r - 1)$ – average dissimilarity of *i*-th object to all other objects of P_r cluster;

$b(i) = \min_{s \neq r}\{d_{iP_s}\}$,

$d_{iP_s} = \sum_{k \in P_s} d_{ik} \Big/ n_s$ – average dissimilarity of *i*-th object to all objects of P_s cluster.

The value of u, which maximizes $S(u)$, is regarded as specifying the number of clusters.

References

Gatnar, E., Walesiak, M. (Eds.) (2004), *Metody statystycznej analizy wielowymiarowej w badaniach marketingowych [Multivariate statistical analysis methods in marketing research]*, Wydawnictwo AE, Wroclaw, 342-343, erratum.

Kaufman, L., Rousseeuw, P.J. (1990), *Finding groups in data: an introduction to cluster analysis*, Wiley, New York, 83-88.

Gordon, A.D. (1999), *Classification*, Chapman & Hall/CRC, London, 204-206.

Rousseeuw, P.J. (1987), *Silhouettes: a graphical aid to the interpretation and validation of cluster analysis*, “Journal of Computational and Applied Mathematics”, 20, 53-65.
