# Supplementary material for: DockAnalyse: an application for the analysis of protein-protein interactions
Source: BMC Struct Biol. 2010 Oct 22;10:37. doi: 10.1186/1472-6807-10-37 (PMC2987812; doi:10.1186/1472-6807-10-37)
Supplement: Additional file 1 — DockAnalyse. 4 R program files, 4 Perl scripts, 2 text files and 3 zippped libraries. [file 1472-6807-10-37-S1.ZIP › clusterSim_0.37-1.zip!/clusterSim/pdf/replicationMod_details.pdf]

replication.Mod(clusterSim)

Modification of replication analysis for cluster validation

Formally, replicating clusters proceeds as follows (see Breckenridge [2000], 262-263; Milligan [1996], 368-369; Gordon [1999], 184; Walesiak [2007]):

1. The data are randomly divided into two samples: sample *A* (arbitrarily primary) and sample *B* (replication). Data must exist on the same set of variables in both samples.
2. Sample *A* is clustered (via `kmeans`, `pam` or any hierarchical agglomerative method: `single`, `complete`, `average`, `mcquitty`, `median`, `centroid`, `Ward`) into *u* clusters $A = \{A_1, \ldots, A_u\}$. Decisions regarding variable normalization, distance measure, and selection of the number of clusters are to be completed. Once clusters have been identified, the centroids of *u* clusters are computed (for metric data) or *u* representative objects (often called centrotypes or medoids) are selected (for metric or nonmetric data).
3. Sample *B* is clustered, using the same clustering procedure, into *u* clusters $B = \{B_1, \ldots, B_u\}$.
4. Determine the distances between sample *B* objects to the centroids (or medoids) of sample *A*. Assign objects from sample *B* to their nearest centroid (or medoid). Each element in sample *B* is assigned to the nearest centroid (or medoid) determined from the sample *A* (this provides a partition of sample *B* into no more than *u* clusters). This produces a clustering of sample *B* based on characteristics of sample *A*: $B^* = \{B_1^*, \ldots, B_u^*\}$.
5. Repeat steps 1-4 *S* times (*S* – the number of simulations).
6. Compute a measure of agreement (mean corrected Rand index) between two clusterings *B* and B^* for *S* simulations. The level of agreement between the two partitions reflects the stability of the clustering in the data.

References

Breckenridge, J.N. (2000), *Validating cluster analysis: consistent replication and symmetry*, "Multivariate Behavioral Research", 35 (2), 261-285.

Gordon, A.D. (1999), *Classification*, Chapman and Hall/CRC, London.

Hubert, L., Arabie, P. (1985), *Comparing partitions*, "Journal of Classification", no. 1, 193-218.

Milligan, G.W. (1996), *Clustering validation: results and implications for applied analyses*, In P. Arabie, L.J. Hubert, G. de Soete (Eds.), *Clustering and classification*, World Scientific, Singapore, 341-375.

Walesiak, M. (2008), *Ocena stabilnosci wynikow klasyfikacji z wykorzystaniem analizy replikacji*, In: J. Pociecha (Ed.), Modelowanie i prognozowanie zjawisk spoleczno-gospodarczych, Wydawnictwo UE, Krakow, 67-72.
