# Supplementary material for: DockAnalyse: an application for the analysis of protein-protein interactions
Source: BMC Struct Biol. 2010 Oct 22;10:37. doi: 10.1186/1472-6807-10-37 (PMC2987812; doi:10.1186/1472-6807-10-37)
Supplement: Additional file 1 — DockAnalyse. 4 R program files, 4 Perl scripts, 2 text files and 3 zippped libraries. [file 1472-6807-10-37-S1.ZIP › fpc_1.2-7.zip!/fpc/html/00Index.html]

R: Fixed point clusters, clusterwise regression and discriminant
plots

# Fixed point clusters, clusterwise regression and discriminant plots


---

## Documentation for package ‘fpc’ version 1.2-7

- DESCRIPTION file.

## Help Pages

|  |  |
| --- | --- |
| adcoord | Asymmetric discriminant coordinates |
| ancoord | Asymmetric neighborhood based discriminant coordinates |
| awcoord | Asymmetric weighted discriminant coordinates |
| batcoord | Bhattacharyya discriminant projection |
| batvarcoord | Bhattacharyya discriminant projection |
| can | Generation of the tuning constant for regression fixed point clusters |
| claraCBI | Interface functions for clustering methods |
| clujaccard | Jaccard similarity between logical vectors |
| clusexpect | Expected value of the number of times a fixed point cluster is found |
| cluster.stats | Cluster validation statistics |
| clusterboot | Clusterwise cluster stability assessment by resampling |
| cmahal | Generation of tuning constant for Mahalanobis fixed point clusters. |
| con.comp | Connectivity components of an undirected graph |
| cov.wml | Weighted Covariance Matrices (Maximum Likelihood) |
| cweight | Weight function for AWC |
| dbscan | Clustering: DBSCAN density reachability and connectivity |
| dbscanCBI | Interface functions for clustering methods |
| discrcoord | Discriminant coordinates/canonical variates |
| discrproj | Linear dimension reduction for classification |
| disthclustCBI | Interface functions for clustering methods |
| distnoisemclustCBI | Interface functions for clustering methods |
| disttrimkmeansCBI | Interface functions for clustering methods |
| fixmahal | Mahalanobis Fixed Point Clusters |
| fixreg | Linear Regression Fixed Point Clusters |
| fpclusters | Extracting clusters from fixed point cluster objects |
| fpclusters.mfpc | Mahalanobis Fixed Point Clusters |
| fpclusters.rfpc | Linear Regression Fixed Point Clusters |
| fpmi | Mahalanobis Fixed Point Clusters |
| hclustCBI | Interface functions for clustering methods |
| hclusttreeCBI | Interface functions for clustering methods |
| itnumber | Number of regression fixed point cluster iterations |
| kmeansCBI | Interface functions for clustering methods |
| kmeansruns | k-means clustering with several random initializations |
| mahalanodisc | Mahalanobis for AWC |
| mahalanofix | Mahalanobis distances from center of indexed points |
| mahalanofuz | Mahalanobis distances from center of indexed points |
| mahalCBI | Interface functions for clustering methods |
| mahalconf | Mahalanobis fixed point clusters initial configuration |
| minsize | Minimum size of regression fixed point cluster |
| mvdcoord | Mean/variance differences discriminant coordinates |
| ncoord | Neighborhood based discriminant coordinates |
| noisemclustCBI | Interface functions for clustering methods |
| pamk | Partitioning around medoids with estimation of number of clusters |
| pamkCBI | Interface functions for clustering methods |
| plot.clboot | Clusterwise cluster stability assessment by resampling |
| plot.dbscan | Clustering: DBSCAN density reachability and connectivity |
| plot.mfpc | Mahalanobis Fixed Point Clusters |
| plot.rfpc | Linear Regression Fixed Point Clusters |
| plotcluster | Discriminant projection plot. |
| predict.dbscan | Clustering: DBSCAN density reachability and connectivity |
| print.clboot | Clusterwise cluster stability assessment by resampling |
| print.dbscan | Clustering: DBSCAN density reachability and connectivity |
| print.mfpc | Mahalanobis Fixed Point Clusters |
| print.rfpc | Linear Regression Fixed Point Clusters |
| print.summary.mfpc | Mahalanobis Fixed Point Clusters |
| print.summary.rfpc | Linear Regression Fixed Point Clusters |
| randcmatrix | Random partition matrix |
| randconf | Generate a sample indicator vector |
| regem | Mixture Model ML for Clusterwise Linear Regression |
| regmix | Mixture Model ML for Clusterwise Linear Regression |
| rFace | "Face-shaped" clustered benchmark datasets |
| rfpi | Linear Regression Fixed Point Clusters |
| simmatrix | Extracting intersections between clusters from fpc-object |
| solvecov | Inversion of (possibly singular) symmetric matrices |
| sseg | Position in a similarity vector |
| summary.mfpc | Mahalanobis Fixed Point Clusters |
| summary.rfpc | Linear Regression Fixed Point Clusters |
| tdecomp | Root of singularity-corrected eigenvalue decomposition |
| tonedata | Tone perception data |
| trimkmeansCBI | Interface functions for clustering methods |
| wfu | Weight function (for Mahalabobis distances) |
